# The oral exposome and environment-related diseases of hard and soft oral tissues: a comprehensive review

**DOI:** 10.3389/froh.2026.1976898

**Published:** 2026-10-27

**Authors:** Angelo Aliberti, Noemi Coppola, Roberta Gasparro, Oreste Trosino, José Camilla Sammartino, Gilberto Sammartino

**Affiliations:** 1 Department of Neurosciences, Reproductive Sciences and Odontostomatological Sciences, University of Naples Federico II, Naples, Italy; 2 Department of Integrated Time-Dependent Network Activities for Stroke, Surgical Emergencies, and Trauma, Head and Neck Outpatient and Inpatient Specialties, Federico II University Hospital, Naples, Italy; 3 Department of Medicine, Surgery and Dentistry, University of Salerno, Salerno, Italy

**Keywords:** bone tissue regeneration, environmental exposure, microbial dysbiosis, oral exposome, oral microbiome, oral mucosal alterations, oral tissue homeostasis, peri-implant disease

## Abstract

**Background:**

The oral cavity is continuously exposed to environmental, lifestyle-related, microbial, and systemic influences capable of altering tissue homeostasis and contributing to chronic oral disease. Environmental exposures may interact through inflammatory, microbial, and metabolic pathways affecting both hard and soft oral tissues. This review critically synthesizes current evidence on environment-related oral diseases within the framework of the oral exposome, focusing on environmental determinants, biological mechanisms, tissue alterations, and implications for oral surgery, implantology, regenerative procedures, and precision oral medicine.

**Materials and methods:**

A comprehensive narrative review was conducted through electronic searches of PubMed, Scopus, and Web of Science for studies published up to April 2026. English-language articles evaluating relationships between environmental exposure and oral tissue alterations were included. Evidence regarding environmental pollutants, smoking, alcohol consumption, dietary factors, occupational exposures, and other external determinants was critically analyzed together with host-related modifiers, including salivary dysfunction, microbial dysbiosis, systemic disease, and chronic inflammatory conditions. The review considered exposure-associated alterations involving dental hard tissues, periodontal and peri-implant structures, oral mucosa, salivary homeostasis, and oral microbial ecology.

**Results:**

The strength of evidence varied substantially across exposure–outcome relationships. The most mature human evidence concerned conventional tobacco smoking and selected carcinogenic exposures, whereas associations involving air pollution, heavy metals, occupational toxicants, electronic cigarettes, endocrine-disrupting chemicals, and emerging contaminants showed greater heterogeneity and, for several outcomes, relied substantially on observational, experimental, or mechanistic evidence. Across these different levels of evidence, recurrent pathways included oxidative stress, inflammation, microbial dysbiosis, epithelial dysfunction, and altered tissue homeostasis.

**Conclusions:**

The oral exposome provides a useful framework for understanding the multifactorial nature of oral disease and the interaction among environmental exposure, microbial ecology, host response, and tissue homeostasis.

## 1 Introduction

The oral cavity represents one of the most environmentally exposed and biologically dynamic anatomical systems of the human body (1). Through breathing, alimentation, hydration, oral hygiene practices, occupational exposure, and lifestyle-related behaviors, oral tissues are continuously subjected to a broad spectrum of physical, chemical, microbial, and climatic stressors (2, 3). This constant interface with the external environment makes the oral cavity particularly responsive to environmental influences. Environmental exposures may interact directly or indirectly with epithelial barriers, saliva, dental hard tissues, periodontal and peri-implant tissues, alveolar bone, immune responses, and the oral microbiome (4, 5).

Oral diseases are among the most prevalent non-communicable conditions worldwide and are associated with major functional, psychosocial, economic, and systemic health consequences. Dental caries, periodontal diseases, tooth loss, oral mucosal disorders, and cancers of the lip and oral cavity continue to impose a substantial global burden, despite major advances in preventive and therapeutic dentistry (6). Traditionally, the pathogenesis of oral diseases has been interpreted mainly through local models centered on microbial biofilm accumulation, host inflammatory response, behavioral habits, and genetic susceptibility (7). Although these factors remain essential, this framework is no longer sufficient to fully explain the complexity, heterogeneity, and progression of several oral diseases affecting both hard and soft tissues.

In recent years, the concept of the exposome has provided a broader framework for understanding chronic disease pathogenesis (8). Originally introduced by Wild, the exposome refers to the totality of environmental exposures experienced throughout life and their biological effects, complementing the genome in explaining disease risk (9). Within oral medicine, this concept has evolved into the framework of the “oral exposome”, defined as the cumulative and dynamic interaction between environmental exposures and the oral ecosystem, including saliva, mucosal surfaces, dental tissues, periodontal structures, alveolar bone, immune networks, and resident microbial communities (10, 11). Importantly, the oral exposome should not be interpreted simply as an expanded catalogue of environmental risk factors. Its distinctive value lies in considering the cumulative, time-dependent, and potentially interacting nature of multiple exposures together with host susceptibility and the biological responses of the oral ecosystem. Inclusion of an exposure within this framework does not, however, imply that a causal relationship with a specific oral outcome has been established. Environmental exposures considered in relation to the oral exposome include external physical, chemical, occupational, and lifestyle-related determinants, such as pollutants, tobacco-related products, alcohol, dietary factors, ultraviolet radiation, and emerging contaminants. Systemic conditions, salivary alterations, and microbial dysbiosis are instead regarded as host-related or biological modifiers that may shape the response of the oral ecosystem to these exposures. This distinction allows external determinants to be considered alongside individual susceptibility without treating them as equivalent components of exposure. Rather than acting as a passive interface, the oral cavity responds to environmental stressors through coordinated inflammatory, immunological, metabolic, and microbiological adaptations (12). Depending on the nature, intensity, duration, and biological context of exposure, persistent environmental stressors may disrupt oral homeostasis and increase tissue vulnerability. The oral microbiome represents an important component of this framework, as environmental exposures may modify host–microbial interactions and local ecological balance (13). These interactions are examined in greater detail within the mechanistic synthesis below.

Air pollution has recently emerged as an important environmental determinant of oral health (14). Growing evidence suggests associations between chronic exposure to airborne pollutants and several oral conditions, particularly periodontal disease, although the underlying biological mechanisms remain incompletely understood (5). Similarly, tobacco smoking remains one of the most extensively documented environmental risk factors for oral pathology, with substantial evidence linking it to periodontal destruction, peri-implant complications, oral potentially malignant disorders, oral squamous cell carcinoma, impaired wound healing, and adverse surgical outcomes (15). The rapid diffusion of electronic cigarettes and heated tobacco products further strengthens the need to reconsider environmental exposure in oral health (16). Preliminary experimental and translational evidence suggests that vaping may influence oral tissues through immune impairment, microbiome alteration, mucosal irritation, periodontal inflammation, and salivary changes, although long-term clinical consequences remain incompletely defined (17).

A further emerging area concerns environmental toxicants beyond tobacco smoke and air pollution. Heavy metals, industrial chemicals, occupational pollutants, and more recently microplastics and nanoplastics have all been associated with oral mucosal alterations, salivary dysfunction, periodontal inflammation, and impaired tissue homeostasis (5, 18, 19).

Climate-related environmental changes may additionally influence salivary function, mucosal vulnerability, infectious susceptibility, and exposure-related oral disease patterns (20). Actinic cheilitis and lip squamous cell carcinoma represent clear examples of oral diseases in which environmental exposure, tissue vulnerability, and malignant potential intersect. Importantly, the influence of environmental factors extends beyond disease onset (21). Environmental determinants may also be relevant to the biological conditions required for oral surgery, implantology, and regenerative treatment, including processes involved in wound healing, osseointegration, and tissue regeneration (22, 23). However, the strength of direct clinical evidence varies considerably among individual exposures and outcomes. Accordingly, surgical and regenerative implications represent an important, but still incompletely characterized, dimension of the oral exposome (10).

Despite increasing interest in environmental health, the evidence linking environmental exposures to oral diseases remains fragmented across dentistry, oral medicine, toxicology, epidemiology, occupational medicine, microbiology, oncology, and environmental sciences. Most available studies focus on single exposures or isolated disease entities, while few attempts have been made to integrate environmental toxicology, oral microbiome alterations, hard and soft tissue pathology, and surgical implications within a unified conceptual framework (24, 25). This area remains insufficiently explored.

Although numerous studies have investigated individual environmental risk factors, an integrated exposomic perspective encompassing both hard and soft oral tissues and their implications for oral medicine, oral surgery, and implantology remains largely lacking (5, 26, 27). Therefore, the present narrative review aims to critically synthesize the current evidence on environment-related diseases affecting hard and soft oral tissues within the framework of the oral exposome. Specifically, it examines the principal environmental determinants implicated in oral diseases, the biological mechanisms through which environmental exposures influence oral tissue homeostasis, the major hard and soft tissue disorders associated with chronic environmental burden, and the implications of these exposures for oral surgery, implantology, wound healing, and regenerative procedures. Finally, the review identifies current knowledge gaps and discusses future perspectives for exposomics, salivary biomarkers, microbiome-based diagnostics, and precision oral medicine.

By integrating mechanistic, epidemiological, clinical, and surgical perspectives, this review aims to provide a comprehensive framework for critically interpreting the relationships between environmental exposures, oral disease susceptibility and progression, and treatment outcomes. Such an approach may provide a basis for future advances in prevention, personalized risk assessment, and more environmentally informed models of oral healthcare, while recognizing that the clinical applicability of these concepts currently varies according to the strength of evidence for individual exposures.

The conceptual framework of the oral exposome and its biological implications for oral tissues is summarized in Figure 1.

**Figure 1:**
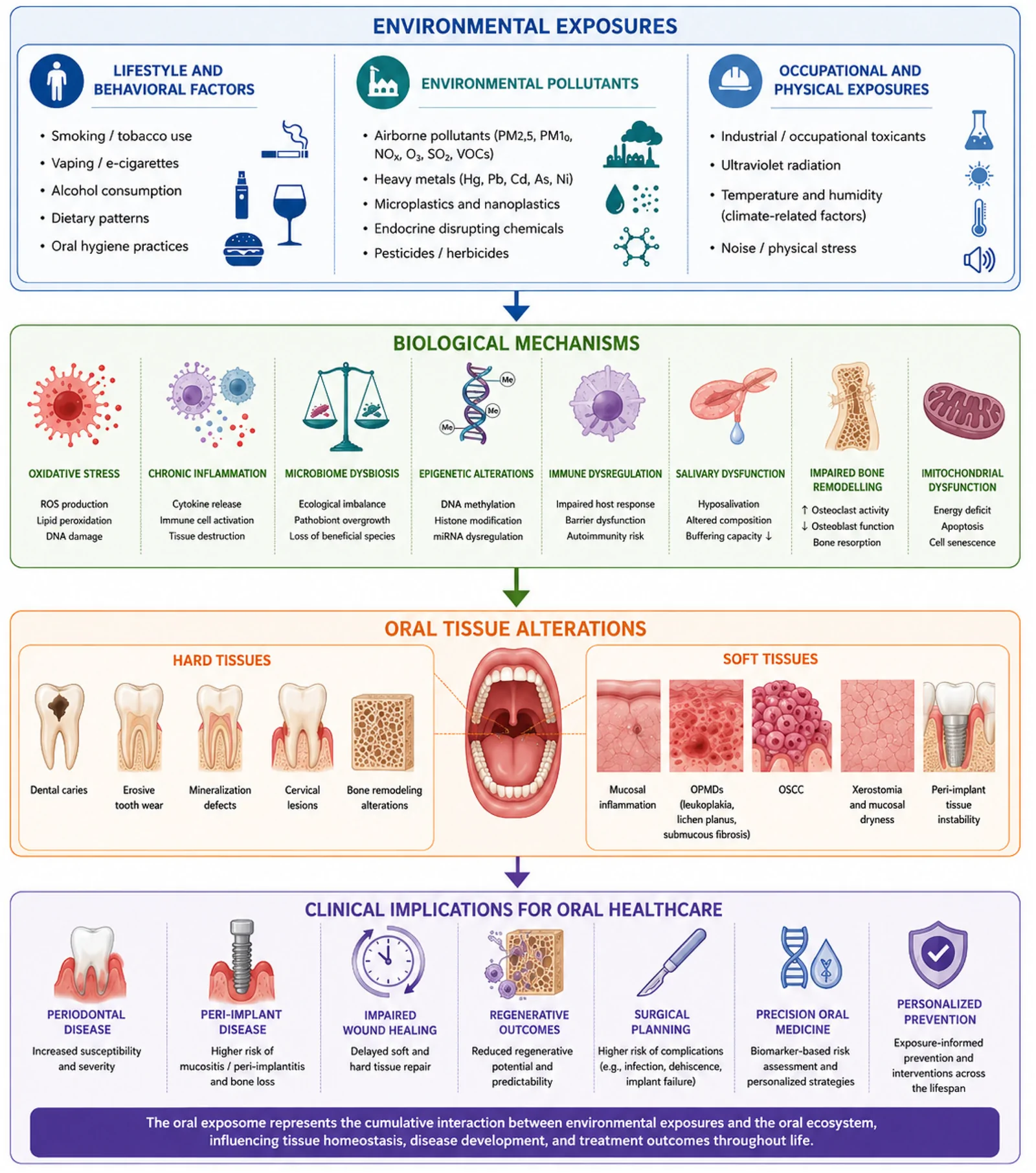
Conceptual framework of the oral exposome. Environmental, lifestyle-related, occupational, and systemic exposures may interact with host and microbial factors through interconnected biological mechanisms influencing oral tissue homeostasis. Depending on the exposure and the strength of the available evidence, these interactions have been associated with alterations of hard and soft oral tissues and may contribute to differences in disease susceptibility, regenerative processes, and clinical outcomes. The pathways illustrated represent a conceptual synthesis and are not supported by equivalent levels of evidence; their evidentiary maturity ranges from established human relationships to emerging or hypothesis-generating mechanisms.

## 2 Materials and methods

### 2.1 Literature search strategy

This narrative review was conducted to provide a clinically oriented synthesis of the current evidence regarding the role of environmental exposures in diseases affecting hard and soft oral tissues. Given the multidisciplinary nature of the topic and the heterogeneity of the available literature, encompassing mechanistic, experimental, translational, epidemiological, and clinical research, a narrative approach was considered the most appropriate methodology to integrate current knowledge within the conceptual framework of the oral exposome. The review was designed to critically integrate evidence on biological mechanisms, clinical manifestations, and translational implications of environmental exposures affecting oral tissues rather than to quantitatively compare study outcomes. Owing to the heterogeneity of the available evidence, no quantitative synthesis or meta-analysis was planned.

A comprehensive literature search was performed using the PubMed/MEDLINE, Scopus, and Web of Science databases from database inception to April 2026. The final search was conducted on April 15, 2026. During manuscript preparation and revision, a limited number of subsequently published articles were additionally consulted to contextualize recent developments in the Discussion; these publications were not included in the formal study-selection process or in the qualitative evidence appraisal. Only articles published in English were considered eligible. The search strategy combined Medical Subject Headings (MeSH) and free-text terms related to environmental exposures, oral diseases, oral microbiology, oral pathology, periodontology, implantology, oral surgery, carcinogenesis, environmental toxicology, salivary dysfunction, wound healing, and regenerative processes.

To maximize the retrieval of relevant evidence, the reference lists of all included articles were manually screened. In addition, targeted hand-searching was performed in leading peer-reviewed journals in the fields of oral medicine, oral pathology, periodontology, implantology, oral surgery, and environmental health, including *Journal of Dental Research*, *Journal of Dentistry*, *Clinical Oral Investigations*, *Oral Diseases*, *Oral Oncology*, *Clinical Oral Implants Research*, *Journal of Clinical Periodontology*, *Periodontology 2000*, *International Journal of Oral Science*, *Environmental Research*, and *Science of the Total Environment*. Particular attention was given to landmark studies, consensus statements, systematic reviews, translational investigations, and recent high-impact publications addressing both established and emerging environmental determinants of oral health and their biological implications for oral tissues. Reference-list screening and targeted hand-searching were additionally used to verify the coverage of relevant landmark and recent publications; these supplementary search procedures were not recorded as a separate numerical stream.

The following search strategy was used for the PubMed/MEDLINE database. Minor modifications were applied to Scopus and Web of Science according to the indexing systems and search functionalities of each database: ((“oral exposome”) OR (“environmental exposure”) OR (“environmental factors”) OR (“air pollution”) OR (“particulate matter”) OR smoking OR tobacco OR vaping OR (“electronic cigarettes”) OR (“heated tobacco products”) OR alcohol OR (“heavy metals”) OR (“occupational exposure”) OR pollutants OR microplastics OR nanoplastics OR pesticides OR (“endocrine disruptors”) OR (“climate change”)) AND ((“oral diseases”) OR (“oral pathology”) OR (“oral microbiome”) OR dysbiosis OR (“periodontal disease”) OR periodontitis OR (“peri-implant disease”) OR peri-implantitis OR (“oral mucosal diseases”) OR (“oral mucosal lesions”) OR (“oral squamous cell carcinoma”) OR (“oral potentially malignant disorders”) OR xerostomia OR (“salivary dysfunction”) OR (“bone remodeling”) OR osseointegration OR (“bone regeneration”) OR (“wound healing”) OR (“oral surgery”) OR implantology).

The search strategy was designed to identify studies addressing both the biological mechanisms and the clinical implications of environmental exposures affecting oral hard and soft tissues, in accordance with the objectives of this narrative review.

### 2.2 Eligibility criteria and study selection

Eligibility criteria were defined *a priori* to ensure a transparent and consistent selection of the literature relevant to the objectives of this narrative review. Considering the multidisciplinary nature of the topic, a broad range of study designs was considered eligible, including *in vitro* and *ex vivo* investigations, animal studies, epidemiological research, observational clinical studies, translational investigations, as well as relevant narrative reviews, systematic reviews, consensus statements, and position papers.

Studies were considered eligible if they investigated one or more of the following aspects: (i) the effects of environmental exposures on oral hard or soft tissues; (ii) associations between environmental determinants and oral diseases, including periodontal diseases, peri-implant diseases, oral mucosal disorders, oral potentially malignant disorders, oral squamous cell carcinoma, salivary dysfunction, bone alterations, impaired wound healing, or regenerative complications; (iii) biological mechanisms underlying environmentally mediated oral tissue injury, including oxidative stress, chronic inflammation, microbiome dysbiosis, immune dysregulation, epigenetic alterations, and impaired bone remodeling; or (iv) the influence of environmental factors on oral surgery, implantology, regenerative procedures, and peri-implant tissue stability.

Studies unrelated to oral tissues or oral diseases, publications focused exclusively on systemic conditions without direct oral relevance, conference abstracts without full-text availability, articles lacking sufficient methodological information, and reports considered outside the scope of the review were excluded.

Titles and abstracts identified through the electronic search were initially screened to identify publications relevant to the objectives of the review. Publications considered potentially relevant subsequently underwent full-text evaluation according to the predefined eligibility criteria. Study selection was independently performed by two reviewers (A.A. and R.G.) to improve the consistency and transparency of the selection process. Any disagreements were resolved through discussion and consensus, with consultation of a third reviewer (G.S.) when necessary.

Preference was given to studies providing biological plausibility, methodological transparency, clinically relevant findings, and translational value, allowing the integration of evidence derived from different experimental, clinical, and epidemiological settings within the framework of the oral exposome. The selected studies were subsequently organized into predefined thematic domains and integrated into the narrative synthesis according to the objectives of the review. The electronic database searches identified 1,284 records. After removal of 367 duplicates, 917 records underwent title and abstract screening. Of these, 671 were excluded because they did not meet the predefined eligibility criteria, leaving 246 articles for full-text assessment. Following full-text evaluation, 63 articles were excluded because they did not fulfill the eligibility criteria or were not sufficiently relevant to the objectives of the review. Overall, 183 publications were included in the final narrative synthesis. The study selection process is summarized in Figure 2.

**Figure 2:**
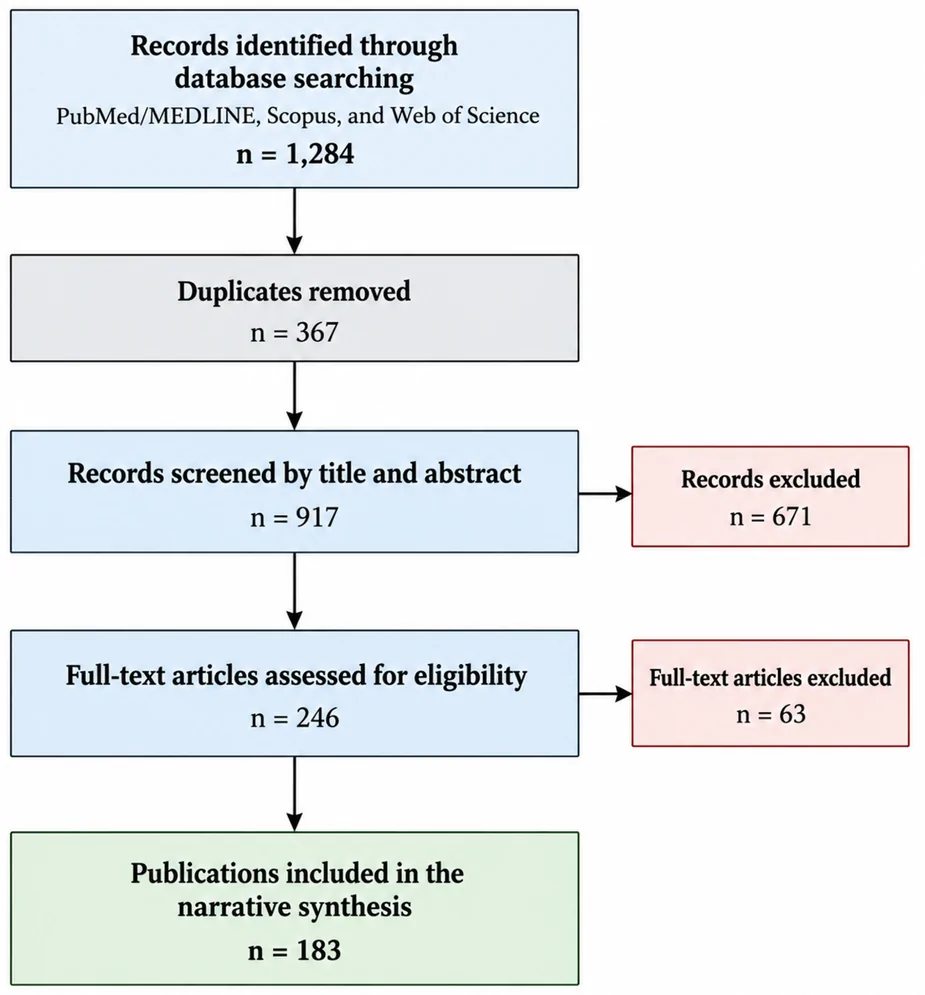
Flow diagram of the literature search and study selection process.

### 2.3 Evidence synthesis and critical appraisal

Given the heterogeneity of the available literature in terms of study design, investigated environmental exposures, biological models, and reported outcomes, a quantitative synthesis was not considered appropriate. Instead, the available evidence was synthesized using a qualitative thematic approach, allowing the integration of mechanistic, experimental, translational, epidemiological, and clinical findings within the conceptual framework of the oral exposome.

Following study selection, the included evidence was organized into predefined thematic domains reflecting the objectives of the review. These domains comprised: (I) environmental exposures and their biological effects; (II) oxidative stress, inflammation, and immune dysregulation; (III) oral microbiome dysbiosis and ecological disruption; (IV) epithelial, connective tissue, and bone alterations; (V) carcinogenic mechanisms and oral potentially malignant disorders; (VI) tissue regeneration, wound healing, and osseointegration; and (VII) the clinical implications of environmental exposures for oral medicine, periodontology, implantology, and oral surgery.

The evidence was critically interpreted according to its biological plausibility, methodological transparency, clinical relevance, translational value, and overall consistency across different study designs. Greater interpretative weight was assigned to evidence derived from systematic reviews, well-designed clinical investigations, epidemiological studies, and reproducible translational research, whereas findings from preliminary experimental and mechanistic studies were interpreted with appropriate caution.

Evidence from different study designs was interpreted according to the type of inference that each design could reasonably support. Experimental and mechanistic studies were primarily used to assess biological plausibility and to identify potential cellular and molecular pathways, whereas observational and epidemiological studies were considered mainly for evidence of exposure–outcome associations in human populations, with attention to potential confounding and limitations in causal inference. Clinical studies were given particular consideration when they provided direct evidence of clinically relevant outcomes, although their interpretation also accounted for study design, sample characteristics, exposure assessment, and consistency with the broader literature. Accordingly, mechanistic plausibility was considered supportive but was not regarded as equivalent to evidence of a clinically established exposure–outcome relationship.

To make differences in evidentiary maturity more explicit, the principal exposure–oral outcome relationships were additionally summarized using a qualitative evidence-strength matrix. The appraisal considered the predominant source of evidence (human clinical/epidemiological, translational, or preclinical/mechanistic), the consistency of findings across studies, the availability of longitudinal or exposure–response data, and the directness of the evidence for the oral outcome considered. On this basis, exposure–outcome relationships were classified as established, supported but predominantly associative, emerging, or hypothesis-generating. These categories were intended to distinguish relationships supported by convergent human evidence from those based mainly on observational associations, experimental findings, or biological plausibility. They should not be interpreted as formal certainty-of-evidence ratings, since a standardized risk-of-bias assessment and GRADE evaluation were outside the scope of this narrative review.

Considering the multidisciplinary scope of the review and the diversity of the included evidence, the application of formal risk-of-bias assessment tools was not considered appropriate. Instead, the critical appraisal focused on the methodological characteristics of the included studies, the consistency of the available evidence, the biological plausibility of the proposed mechanisms, and their potential clinical applicability. This approach allowed the integration of heterogeneous sources of evidence into a coherent narrative synthesis while maintaining a critical interpretation of the current knowledge regarding the role of environmental determinants in the development, progression, and clinical management of diseases affecting hard and soft oral tissues.

## 3 Results

The available evidence suggests that environment-related oral diseases may arise through complex interactions among environmental stressors, host responses, microbial ecology, and tissue susceptibility rather than through simple, isolated exposure–disease relationships (28). Across the included literature, oxidative stress, chronic inflammation, microbial dysbiosis, epithelial dysfunction, immune dysregulation, and altered bone remodeling recurrently emerged as biological pathways potentially linking environmental exposures with oral tissue alterations (29).

The strength and maturity of these relationships, however, varied substantially across exposures and outcomes. Tobacco-related exposures were supported by the most extensive human evidence, particularly for periodontal destruction, oral carcinogenesis, impaired wound healing, and implant-related complications (30, 31). For air pollution, heavy metals, occupational toxicants, and emerging environmental contaminants, the available evidence was more heterogeneous and included varying combinations of observational, experimental, and mechanistic findings (5, 30, 31). Similarly, although environmental burden has been proposed to influence wound healing, osseointegration, peri-implant tissue stability, and regenerative outcomes, the extent of direct clinical evidence differed considerably according to the exposure considered (27). These differences in evidentiary maturity are summarized in Table 3, which provides an overview of the principal exposures and oral outcomes, the predominant type of evidence, the main findings from human studies, key limitations and uncertainties, and the corresponding qualitative evidence interpretation.

For clarity of presentation, the available evidence is synthesized according to a pathophysiological and clinically oriented framework. The following sections examine the principal biological mechanisms underlying exposure-associated oral tissue injury, followed by the major hard and soft tissue diseases associated with chronic environmental burden and their implications for oral surgery, regenerative dentistry, and precision oral medicine.

### 3.1 The oral exposome and pathophysiological mechanisms

The oral exposome provides a conceptual framework for interpreting environment-related oral diseases as the consequence of cumulative and interacting environmental exposures rather than isolated environmental insults (10). Within the oral cavity, environmental stressors dynamically interact with epithelial barriers, saliva, periodontal and peri-implant tissues, alveolar bone, immune responses, and resident microbial communities through interconnected inflammatory, oxidative, microbial, metabolic, and regenerative pathways (32).

The biological response to environmental exposure involves closely interconnected mechanisms rather than independent pathways. Oxidative and inflammatory responses may interact with immune dysregulation and altered host–microbiome homeostasis (33–40), while persistent exposure may also affect molecular regulation, epithelial integrity, and tissue repair (41–45). Together, these processes may influence tissue susceptibility, disease progression, and reparative responses (46). Their individual and overlapping contributions are examined in the following subsections. The principal biological mechanisms linking environmental exposures to oral tissue alterations within the framework of the oral exposome are summarized in Figure 3.

**Figure 3:**
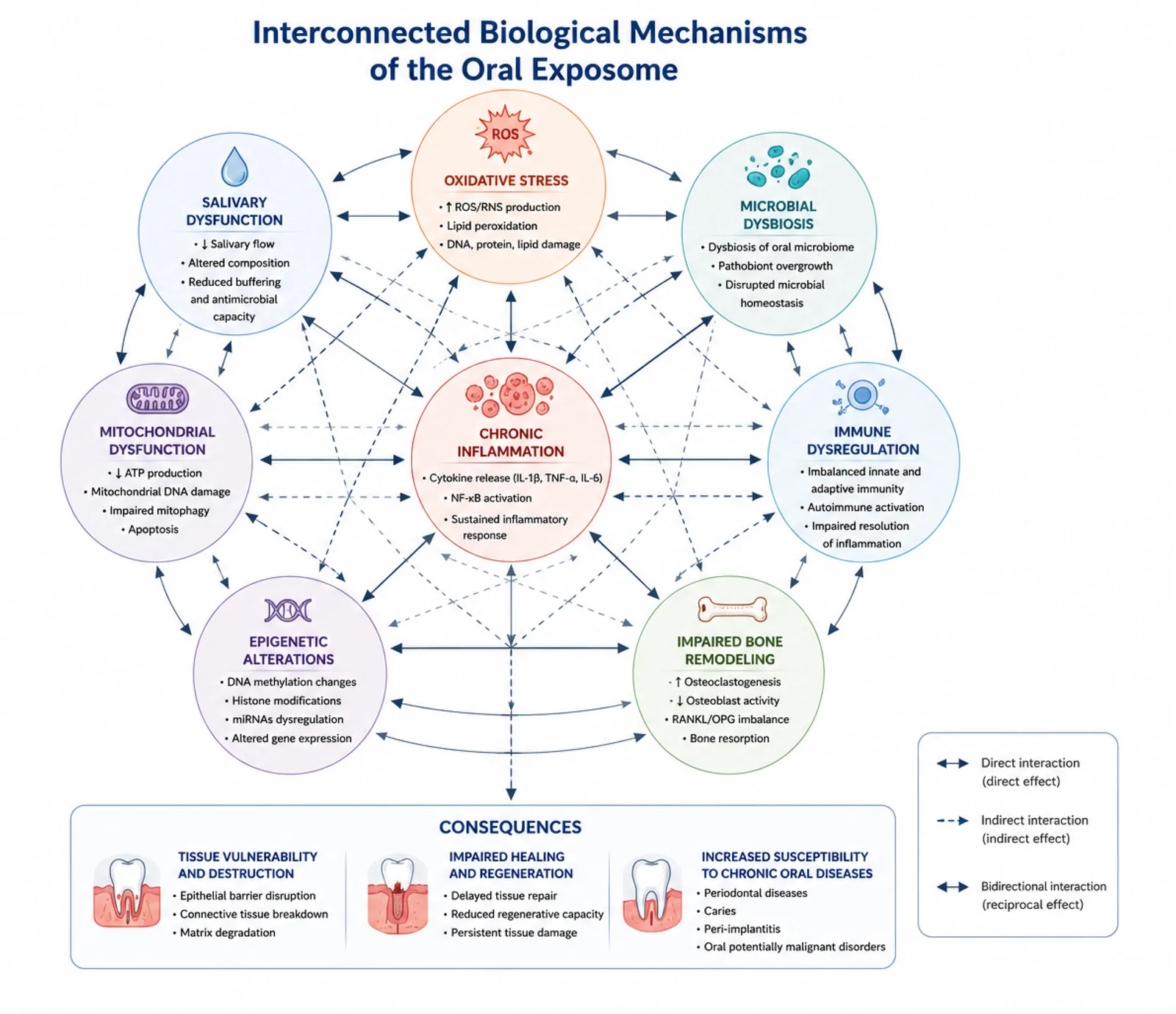
Interconnected biological mechanisms linking environmental exposure to oral tissue alterations within the framework of the oral exposome. Oxidative stress, chronic inflammation, microbial dysbiosis, immune dysregulation, salivary dysfunction, mitochondrial impairment, epigenetic alterations, and altered bone remodeling may interact and contribute to tissue vulnerability, impaired healing, and susceptibility to chronic oral diseases. The figure integrates mechanisms supported by different levels of evidence and should therefore be interpreted as a conceptual framework rather than as implying equivalent causal or clinical validation for all pathways. Evidence ranges from well-established mechanisms for selected exposures to emerging or hypothesis-generating relationships.

#### 3.1.1 Oxidative stress and mitochondrial dysfunction

Altered redox homeostasis represents one of the most extensively investigated mechanisms through which environmental determinants may influence oral tissue integrity (47). Because the oral cavity is continuously exposed to inhaled, ingested, occupational, and lifestyle-related agents, oral tissues experience a persistent pro-oxidative burden that may gradually overwhelm endogenous antioxidant defenses (48). This phenomenon is particularly evident following chronic exposure to tobacco-derived compounds, airborne particulate matter, heavy metals, industrial toxicants, ultraviolet radiation, and electronic cigarette aerosols, all of which may disrupt cellular redox equilibrium through partially overlapping biological pathways.

Under physiological conditions, reactive oxygen species (ROS) and reactive nitrogen species (RNS) participate in several essential biological processes, including intracellular signaling, antimicrobial defense, epithelial turnover, angiogenesis, and tissue repair (49). However, excessive or persistent ROS generation shifts redox signaling from physiological regulation toward pathological tissue injury. Within the oral cavity, this transition appears to result not only from direct toxic exposure but also from the interaction between chronic inflammation, microbial dysbiosis, endothelial dysfunction, and impaired mitochondrial activity (50).

Mitochondria occupy a central role in this process. Besides representing the principal intracellular source of ROS generation, they are highly susceptible to oxidative injury. Experimental evidence indicates that several environmental toxicants may interfere with mitochondrial respiratory complexes, disrupt membrane potential, impair oxidative phosphorylation, and reduce ATP synthesis (51). These alterations progressively compromise cellular metabolism and affect the viability and function of oral epithelial cells, gingival fibroblasts, periodontal ligament cells, osteoblasts, endothelial cells, and salivary gland tissues (52).

Within periodontal and peri-implant tissues, redox dysregulation represents a major pathogenic mechanism. Neutrophils activated by dysbiotic biofilms physiologically generate ROS as part of the host antimicrobial response; however, environmental factors capable of increasing systemic or local oxidative burden may amplify this response and contribute to collateral tissue damage (23, 39). Accordingly, ROS-mediated injury is increasingly recognized not merely as a secondary consequence of inflammation but as an active contributor to extracellular matrix degradation, osteoclastic activation, connective tissue breakdown, and periodontal attachment loss (23).

Tobacco smoke remains one of the clearest examples of environmentally mediated redox injury within the oral cavity. Cigarette smoke contains a complex mixture of oxidants, aldehydes, free radicals, and carcinogenic compounds capable of directly damaging epithelial and connective tissues (53). Beyond its direct cytotoxic effects, smoking may impair antioxidant defense systems, alter neutrophil activity, reduce vascular perfusion, and amplify inflammatory signaling pathways (54). Similar concerns have emerged regarding electronic cigarettes and heated tobacco products. Although their long-term biological effects remain incompletely understood, experimental evidence suggests that vaping aerosols may induce redox imbalance, mitochondrial dysfunction, and inflammatory responses in oral epithelial and periodontal cells even in the absence of combustion-derived products (55).

Comparable biological alterations have also been associated with chronic exposure to air pollution. Fine and ultrafine particulate matter may penetrate biological barriers, stimulate intracellular ROS generation, activate redox-sensitive inflammatory pathways, and interfere with endothelial and mitochondrial function (56). Although much of the available evidence originates from pulmonary and cardiovascular research, growing evidence indicates that particulate exposure may also impair gingival fibroblast viability, compromise epithelial integrity, and promote inflammatory responses within periodontal tissues (57).

Heavy metals provide an additional example of environmentally mediated ROS-driven injury. Cadmium, mercury, arsenic, chromium, and nickel may interfere with antioxidant enzymatic systems, calcium homeostasis, mitochondrial respiration, and DNA repair pathways (58). Their biological effects are often cumulative and may persist over prolonged periods, particularly in chronically exposed populations. Consequently, metal-induced oxidative alterations may affect oral mucosal and periodontal biology and have also been implicated in disturbances of bone metabolism (59). Their potential consequences for oral wound healing, regenerative capacity, and osseointegration remain biologically plausible but require further direct clinical investigation.

The biological consequences of prolonged oxidative stress extend beyond inflammatory tissue injury and include epithelial carcinogenesis. Excessive ROS activity may induce DNA strand damage, lipid peroxidation, telomere instability, and abnormalities in cell-cycle regulation (60). In chronically exposed oral epithelial tissues, these processes may progressively contribute to genomic instability and malignant transformation, representing a mechanistic link between chronic inflammation and oral carcinogenesis.

Altered oxidative signaling may also influence regenerative and surgical outcomes. Physiological ROS activity is required for wound healing, angiogenesis, fibroblast migration, collagen deposition, and bone remodeling (61). Conversely, excessive oxidative burden may impair these processes by disrupting vascular function, reducing osteoblastic differentiation, altering extracellular matrix synthesis, and sustaining chronic inflammatory activation. These mechanisms provide a plausible biological basis for potential alterations in healing, graft maturation, osseointegration, and peri-implant tissue stability (62); however, their clinical relevance depends on the specific exposure and remains insufficiently characterized for several environmental determinants.

Despite growing evidence supporting the role of oxidative stress and mitochondrial dysfunction in environment-related oral diseases, several limitations remain. Many available studies rely on isolated experimental exposure models that only partially reproduce the cumulative, multifactorial, and long-term nature of real-world environmental conditions (63). Furthermore, considerable heterogeneity persists regarding exposure assessment, oxidative stress biomarkers, biological models, and clinical outcome measures, limiting direct comparisons across studies.

#### 3.1.2 Chronic inflammation and immune dysregulation

Among the mechanisms linking chronic environmental burden to oral disease progression, persistent inflammatory dysregulation appears to play a central role. Although inflammatory responses are essential for host defense and tissue repair, sustained immune dysregulation may progressively shift oral tissues toward chronic inflammatory injury (44).

Chronic exposure to tobacco products, airborne pollutants, industrial toxicants, and heavy metals may disrupt inflammatory homeostasis through sustained activation of pro-inflammatory signaling pathways (64). This phenomenon is particularly relevant in periodontal and peri-implant tissues, where tissue destruction frequently reflects an exaggerated or poorly regulated host response rather than the direct effects of microbial challenge alone (39).

Several environmental stressors have been shown to activate redox-sensitive transcription pathways involved in chronic inflammation, including nuclear factor kappa B (NF-*κ*B), activator protein-1 (AP-1), and inflammasome-associated signaling mechanisms (65). The resulting increase in inflammatory mediators, including interleukin-1β, interleukin-6, tumor necrosis factor-α, prostaglandins, and matrix metalloproteinases, may contribute to connective tissue degradation, vascular dysfunction, osteoclastic activation, and epithelial injury (66).

Environmental stressors may also directly influence immune cell function. Tobacco smoke, particulate matter, and several industrial pollutants have been associated with altered neutrophil chemotaxis, impaired phagocytic activity, dysregulated macrophage polarization, and disturbances in T-cell responses (67). Within periodontal tissues, these alterations may compromise microbial clearance while simultaneously sustaining chronic inflammatory activation, thereby promoting progressive connective tissue destruction (68). Similar mechanisms have also been proposed in peri-implant diseases, where persistent inflammatory infiltrates and altered immune responses may contribute to peri-implant bone loss and impaired peri-implant soft tissue stability (69).

Recent evidence suggests that chronically inflamed tissues may also exhibit defective inflammatory resolution (70). Physiological healing requires a coordinated transition from pro-inflammatory signaling toward reparative and pro-resolving pathways. Chronic exposure to smoking, environmental pollutants, and systemic inflammatory burden may interfere with this transition by sustaining cytokine production, endothelial activation, low-grade immune stimulation, and altered tissue metabolism (71, 72). Consequently, prolonged inflammatory activation may contribute to delayed wound healing, impaired regenerative responses, and increased susceptibility to recurrent periodontal and peri-implant inflammation.

The interaction between chronic environmental exposure and immune dysregulation appears particularly relevant in individuals with pre-existing inflammatory susceptibility (73). Systemic conditions characterized by chronic low-grade inflammation, including obesity, metabolic disorders, and cardiovascular disease, may synergistically interact with environmental stressors, promoting periodontal destruction and impairing tissue repair (74). Within this context, the oral exposome reflects the dynamic interplay among environmental burden, systemic inflammatory status, microbial ecology, and individual host susceptibility (1).

Persistent inflammatory activation may also contribute to epithelial transformation and carcinogenesis (75). Continuous cytokine production, immune cell infiltration, altered tissue remodeling, and chronic epithelial injury may generate a microenvironment that favors genomic instability, abnormal cellular proliferation, and resistance to apoptosis (76). In oral potentially malignant disorders and oral squamous cell carcinoma, inflammatory signaling is increasingly recognized not merely as a secondary consequence of neoplastic transformation but as an active contributor to tumor initiation and progression (77). Environmental carcinogens may therefore exert part of their pathogenic effects indirectly through chronic inflammatory and immune-mediated mechanisms.

Persistent inflammatory dysregulation may also compromise reparative responses following oral surgery or implant placement by interfering with vascular, connective-tissue, and osteoimmune homeostasis. This appears particularly relevant in smokers and chronically exposed individuals, in whom altered inflammatory responses have been associated with impaired healing and peri-implant complications (78). Overall, chronic inflammatory dysregulation represents an important pathway through which environmental exposures may modify host responses and tissue susceptibility.

#### 3.1.3 Oral microbiome dysbiosis and ecological disruption

The oral microbiome represents a highly dynamic interface between the host and the external environment. Chronic environmental stressors may progressively disrupt microbial ecological balance through alterations in salivary composition, immune surveillance, epithelial integrity, and local physicochemical conditions, thereby promoting dysbiotic shifts associated with inflammation and increased tissue vulnerability (3).

Environmental exposures may progressively influence microbial community composition, metabolic activity, interspecies interactions, and biofilm architecture, reducing ecological resilience and favoring chronic inflammatory conditions (79). Tobacco exposure represents one of the best-characterized modifiers of oral microbial ecology. Both smoking and smokeless tobacco products have been associated with significant alterations in the composition and metabolic activity of oral biofilms (80). Smokers frequently exhibit increased colonization by anaerobic and proteolytic bacterial species commonly associated with periodontal destruction, together with a reduced prevalence of health-associated commensal taxa (81). Importantly, smoking-associated dysbiosis appears to involve both direct microbial effects and broader ecological alterations affecting host–microbiome interactions. Tobacco-derived compounds may modify oxygen tension, nutrient availability, salivary flow, epithelial permeability, and immune surveillance, thereby creating conditions that favor the establishment and persistence of pathogenic biofilms (82).

Emerging evidence suggests that electronic cigarette aerosols may also influence microbial adhesion, biofilm architecture, and inflammatory microbial profiles, although their long-term ecological consequences remain incompletely understood (83). Available evidence also suggests that vaping may be associated with alterations in periodontal and microbial homeostasis, although the underlying mechanisms and long-term clinical consequences remain incompletely characterized (84). These observations are particularly relevant considering the increasing use of electronic cigarettes among younger populations and the limited knowledge regarding their long-term biological effects on the oral ecosystem.

Air pollution may induce comparable ecological alterations through different exposure pathways. Particulate matter and airborne pollutants may modify epithelial surfaces, salivary composition, local oxidative conditions, and immune responses, thereby indirectly influencing microbial selection within the oral cavity (85). At the population level, prolonged exposure to air pollution has also been associated with an increased risk of periodontal disease (86), although the extent to which alterations of the oral microbiome mediate this relationship remains insufficiently characterized. Nevertheless, methodological variability remains considerable, particularly regarding exposure assessment, microbiome sequencing techniques, and interpretation of ecological changes.

A central feature of dysbiosis is the bidirectional relationship between microbial imbalance and the host inflammatory response. Dysbiotic microbial communities may sustain immune activation through continuous stimulation of epithelial and immune cells, whereas chronic inflammation may further reshape the microbial ecosystem by altering nutrient availability, tissue metabolism, and local oxygen gradients (87). Consequently, microbial dysregulation and chronic inflammation may reinforce each other through self-perpetuating pathogenic interactions (39).

The clinical implications of dysbiosis extend beyond periodontal inflammation. Altered microbial communities are well recognized in peri-implant diseases (88) and have also been investigated in several other oral pathological conditions, including mucosal and neoplastic disorders. Several studies have proposed that persistent microbial imbalance may contribute to epithelial transformation through chronic inflammatory stimulation, production of carcinogenic metabolites, activation of oncogenic signaling pathways, and disruption of epithelial barrier integrity (89). Although definitive causal relationships remain difficult to establish, accumulating evidence supports the concept that ecological disruption of the oral microbiota represents one of the biological mechanisms through which chronic environmental burden can contribute to malignant transformation within the oral cavity.

Beyond direct microbial effects, alterations in salivary homeostasis may further contribute to dysbiotic shifts. Saliva plays a fundamental role in regulating oral pH, buffering capacity, antimicrobial defense, microbial clearance, and nutrient distribution (90). Environmental and host-related factors capable of altering salivary flow or composition may also influence microbial selection and salivary microbial ecology (90–92). Smoking represents one documented example of an exposure associated with salivary microbiome dysbiosis (91). Consequently, salivary dysfunction may simultaneously favor microbial dysbiosis, epithelial vulnerability, caries susceptibility, and impaired tissue repair.

#### 3.1.4 Epigenetic alterations, genotoxicity, and carcinogenic pathways

Beyond their inflammatory and ecological consequences, chronic environmental exposures may induce long-lasting molecular alterations capable of modifying gene regulation, cellular differentiation, and epithelial stability (41). Several toxic agents implicated in oral disease, including tobacco-derived compounds, airborne pollutants, heavy metals, ultraviolet radiation, pesticides, and industrial solvents, have been shown to exert part of their pathogenic activity through epigenetic dysregulation and genotoxic injury (5). Epigenetic dysregulation is increasingly recognized as an important component of oral carcinogenesis and has been investigated in both oral potentially malignant disorders and oral squamous cell carcinoma (93).

Unlike direct mutational events, epigenetic modifications may alter cellular behavior without changing the underlying DNA sequence (94). DNA methylation abnormalities, histone modifications, chromatin remodeling, and altered microRNA expression have all been implicated in exposure-related epithelial dysfunction. These molecular alterations may persist even after exposure reduction, potentially contributing to prolonged tissue susceptibility and abnormal epithelial responses (95). Within the oral cavity, epigenetic regulation is particularly relevant because oral tissues are repeatedly exposed to environmental, microbial, and lifestyle-related influences capable of modifying cellular homeostasis (96).

Tobacco exposure remains the most extensively investigated example of chronic exposure-induced epigenetic and genotoxic damage in oral tissues. Cigarette smoke contains a complex mixture of carcinogens, aldehydes, nitrosamines, polycyclic aromatic hydrocarbons, and reactive radicals capable of inducing DNA adduct formation, oxidative nucleotide injury, chromosomal instability, and aberrant methylation profiles (97). These alterations interfere with apoptosis, cell-cycle regulation, epithelial differentiation, and DNA repair pathways. Importantly, smoking-associated molecular damage appears to accumulate progressively, particularly within chronically exposed mucosal sites, thereby facilitating the transition from epithelial dysplasia to invasive carcinoma (97).

Emerging evidence indicates that airborne pollutants can induce comparable molecular disturbances through partially overlapping biological mechanisms. Fine particulate matter and combustion-derived toxicants have been associated with oxidative DNA damage, epigenetic alterations, and activation of molecular pathways involved in malignant transformation in several tissues (98). Although direct evidence involving the oral mucosa remains more limited than that available for pulmonary disease, increasing attention has focused on the possibility that prolonged exposure to polluted environments may contribute to epithelial vulnerability and carcinogenic susceptibility within the oral cavity (5).

Heavy metals provide another important example of cumulative molecular toxicity. Cadmium, arsenic, chromium, mercury, and nickel are known to interfere with DNA repair systems, mitochondrial metabolism, antioxidant enzymes, and epigenetic regulation (99). In addition to inducing oxidative DNA damage, several metals have demonstrated the ability to alter gene expression profiles involved in inflammation, apoptosis, angiogenesis, and cellular proliferation. A distinctive characteristic of metal-associated toxicity is its cumulative nature, whereby chronic low-dose exposure may progressively induce durable molecular alterations even in the absence of clinically evident toxicity (100).

The interaction between chronic inflammation and genotoxic injury appears particularly relevant in oral carcinogenesis. Chronically inflamed tissues gradually develop a biologically unstable microenvironment characterized by continuous cytokine release, altered epithelial turnover, oxidative nucleotide damage, and defective apoptotic regulation. This concept is particularly relevant to oral potentially malignant disorders, in which chronic inflammatory processes, microbial dysbiosis, and progressive molecular alterations have all been investigated as potential contributors to malignant transformation (101). Epigenetic regulation may also influence regenerative and reparative responses within oral tissues (102). Experimental and mechanistic evidence indicates that environmental toxicants can induce persistent epigenetic alterations with potential consequences for cellular homeostasis and tissue responses (103). However, whether these mechanisms translate into clinically relevant impairment of healing or regeneration following oral surgical and implant procedures remains insufficiently established.

Chronic ultraviolet exposure induces DNA photoproduct formation, oxidative injury, mutations involving tumor-suppressor pathways, and local immunosuppression, thereby promoting photocarcinogenesis (105). In the lip vermilion, cumulative ultraviolet exposure is a major etiological factor underlying actinic cheilitis and its potential progression toward lip squamous cell carcinoma (104).

Collectively, the available evidence supports an important role for epigenetic dysregulation and genotoxic injury in selected exposure-related carcinogenic pathways, although the strength of evidence differs substantially among individual environmental determinants. These mechanisms are well characterized for established carcinogenic exposures, whereas their direct contribution to oral carcinogenesis remains less clearly defined for several emerging pollutants and toxicants. As molecular profiling technologies continue to evolve, integrating epigenetic biomarkers with exposomic assessment may provide a more comprehensive understanding of individual susceptibility to environment-related oral diseases.

#### 3.1.5 Impaired bone remodeling, tissue regeneration, and osseointegration

Bone remodeling within oral tissues depends on a tightly regulated balance between osteoblastic bone formation and osteoclastic resorption (106). This equilibrium is coordinated through complex molecular networks involving receptor activator of nuclear factor kappa-B ligand (RANKL), osteoprotegerin (OPG), inflammatory mediators, growth factors, vascular signaling, and mechanical stimulation (107). Experimental, translational, and clinical findings suggest that selected environmental exposures may interact with pathways regulating bone remodeling through mechanisms involving redox dysregulation, endothelial injury, mitochondrial dysfunction, and persistent inflammatory activation (108). The clinical significance of these effects, however, varies considerably according to the exposure considered. Whereas smoking-related impairment of healing and implant outcomes is supported by comparatively mature human evidence, the effects of several pollutants and emerging contaminants on oral bone quality and regenerative outcomes remain predominantly mechanistic or preclinical.

From an oral surgery perspective, the biological environment of the surgical site may influence postoperative tissue repair. Clinical strategies aimed at modulating this local regenerative environment have therefore attracted increasing interest; randomized clinical evidence has, for example, evaluated injectable platelet-rich fibrin as an adjunct for improving postoperative recovery and healing following impacted mandibular third-molar extraction (109). Environmental stressors capable of disrupting these biological processes may therefore influence regenerative biology, although direct demonstration of clinically relevant impairment remains exposure-dependent.

Smoking remains one of the clearest clinical examples of exposure-related impairment of oral regenerative biology. Tobacco-derived compounds have been associated with reduced vascular perfusion, impaired fibroblast proliferation, altered collagen synthesis, decreased osteoblastic activity, and delayed epithelial repair (110). Nicotine and combustion-related toxicants also interfere with angiogenic signaling and reduce oxygen availability within healing tissues, thereby compromising regenerative dynamics following oral surgery, periodontal therapy, bone augmentation procedures, and implant placement (111). These mechanisms are consistent with the well-established association between smoking and increased rates of periodontal breakdown, implant complications, impaired osseointegration, and delayed postoperative healing (112).

Comparable biological alterations have also been described in relation to airborne pollutants and particulate exposure. Experimental evidence indicates that fine particulate matter and other air pollutants may interfere with osteogenic activity, bone remodeling, and molecular pathways involved in skeletal homeostasis (113). Although direct evidence involving oral regenerative procedures remains limited, these findings support the hypothesis that prolonged exposure to polluted environments may adversely affect alveolar bone metabolism and healing responses.

The interaction between chronic inflammatory activation and bone remodeling appears particularly important in periodontal and peri-implant diseases (114). Persistent inflammatory signaling stimulates osteoclastogenesis through dysregulation of the RANK/RANKL/OPG axis, thereby promoting progressive alveolar bone resorption. Environmental stressors capable of sustaining chronic inflammation may further amplify osteoclastic activity while simultaneously impairing reparative osteoblastic responses (44). Within peri-implant tissues, this imbalance may contribute not only to inflammatory bone loss but also to reduced implant stability and compromised long-term peri-implant tissue health.

Heavy metals and industrial toxicants provide another example of environmentally mediated impairment of regenerative biology. Heavy metals, including cadmium, arsenic, mercury, and lead, can interfere with cellular and metabolic pathways relevant to bone homeostasis (115). Experimental evidence specifically demonstrates that lead and cadmium can exert direct cytotoxic and redox-mediated effects on human osteoblasts (116).

Another emerging area of interest concerns the interaction between implant surface characteristics, epigenetic regulation, and the biological processes underlying osseointegration (117). Sustained inflammatory microenvironments may modify peri-implant immune responses, biofilm ecology, and tissue integration surrounding biomaterials. Although the underlying mechanisms remain incompletely understood, accumulating evidence suggests that chronic oxidative and inflammatory conditions influence soft tissue sealing, peri-implant bone remodeling, and susceptibility to peri-implant disease (118).

The biological consequences of chronic environmental exposure are not restricted to mineralized tissues. Successful oral wound healing also depends on coordinated epithelial migration, fibroblast activity, extracellular matrix deposition, vascular maturation, and timely resolution of inflammation (78). Among environmental exposures, smoking represents the best-documented clinical example of impaired oral wound healing and regenerative outcomes (110–112). Other environmental pollutants and toxicants may interfere with several of the same biological pathways; however, direct clinical evidence linking these exposures to wound dehiscence, graft failure, or impaired oral regenerative outcomes remains limited.

The principal environmental exposures associated with oral tissue alterations and their biological and clinical implications are summarized in Table 1.

**Table 1:** Principal environmental exposures associated with oral tissue alterations and their biological and clinical implications within the framework of the oral exposome.

Environmental exposure	Predominant biological mechanisms	Main affected oral tissues	Reported or potential clinical implications
Tobacco-related products (conventional cigarettes, electronic cigarettes, smokeless tobacco)	Oxidative stress, chronic inflammatory activation, endothelial dysfunction, microbiome dysbiosis, impaired immune regulation	Periodontal tissues, peri-implant tissues, oral mucosa, salivary glands	Periodontitis, peri-implantitis, delayed wound healing, impaired osseointegration, implant instability, oral squamous cell carcinoma
Alcohol exposure	Epithelial barrier disruption, acetaldehyde-mediated toxicity, salivary dysfunction, mucosal barrier impairment	Oral mucosa, salivary glands, periodontal tissues	Oral mucosal lesions, xerostomia, increased carcinogenic susceptibility, periodontal inflammation
Air pollution (particulate matter and gaseous pollutants)	Oxidative stress, endothelial dysfunction, epithelial injury, chronic inflammatory activation, immune dysregulation	Oral mucosa, periodontal tissues, alveolar bone	Periodontal disease; potential impairment of wound healing and regenerative responses; alveolar bone alterations
Heavy metals and industrial toxicants	Genotoxicity, mitochondrial dysfunction, oxidative stress, altered cellular signaling, epigenetic dysregulation	Oral epithelium, alveolar bone, periodontal tissues	Epithelial dysplasia and chronic tissue toxicity; potential impairment of regenerative responses; increased tissue vulnerability
Endocrine-disrupting chemicals	Hormonal imbalance, altered osteoblastic activity, immune modulation, metabolic dysregulation	Alveolar bone, periodontal tissues, peri-implant tissues	Altered bone remodeling; potential periodontal and peri-implant effects; possible impairment of osseointegration and tissue healing
Occupational exposures (chemical and industrial agents)	Oxidative stress, epithelial injury, inflammatory activation, endothelial dysfunction, impaired reparative responses	Oral mucosa, periodontal tissues, salivary glands	Chronic mucosal alterations; potential effects on wound healing; increased tissue vulnerability
Ultraviolet radiation	DNA damage, oxidative injury, epithelial dysregulation, local immune suppression	Lip vermilion epithelium, oral mucosa	Actinic cheilitis, epithelial dysplasia, lip squamous cell carcinoma
Dietary and metabolic factors	Ecological imbalance, salivary dysfunction, inflammatory stress, altered mineral homeostasis	Dental hard tissues, oral microbiome, periodontal tissues	Dental caries, microbial dysbiosis, periodontal inflammation, erosive tooth wear
Microplastics and nanoplastics	Oxidative stress, epithelial injury, inflammatory dysregulation, potential endocrine and immune alterations	Oral mucosa, salivary glands, periodontal tissues	Potential microbial dysbiosis, epithelial vulnerability, inflammatory alterations, and impaired tissue homeostasis

The clinical implications summarized in this table encompass relationships supported by different levels of evidence and should not be interpreted as having equivalent evidentiary maturity. Potential or emerging relationships are explicitly identified where direct clinical evidence remains limited. A qualitative appraisal of evidence strength for the principal exposure–outcome relationships is provided in Table 3.

### 3.2 Environment-related diseases of oral soft tissues

Oral soft tissues represent a direct interface with environmental exposures and may be affected by tobacco-related products, alcohol, occupational agents, airborne pollutants, ultraviolet radiation, and other environmental stressors (5, 119). Reported manifestations range from inflammatory and reactive mucosal alterations to potentially malignant and malignant disorders, although the strength of evidence differs substantially among individual exposures and outcomes. Heavy metals, industrial chemicals, combustion-derived products, and airborne pollutants have also been associated with mucosal alterations through local or systemic exposure pathways (25). In occupationally or chronically exposed populations, careful assessment of exposure history may therefore provide useful context during the evaluation of oral mucosal abnormalities (120, 121).

#### 3.2.1 Chronic inflammatory and reactive oral mucosal disorders

Chronic inflammatory and reactive disorders of the oral mucosa represent some of the most frequent soft tissue manifestations associated with prolonged environmental exposure (5). Although these lesions are often multifactorial in origin, accumulating evidence indicates that sustained exposure to tobacco-derived compounds, airborne pollutants, occupational irritants, alcohol consumption, chronic mechanical trauma, and chemical agents may impair epithelial resilience and local immune regulation, thereby increasing susceptibility to persistent mucosal injury (19). In many cases, lesion development appears to reflect the cumulative effects of repeated epithelial damage and defective inflammatory resolution rather than direct toxic injury alone.

Tobacco-associated mucosal alterations remain among the clearest examples of exposure-related soft tissue injury within the oral cavity. Chronic smoking has been associated with epithelial hyperkeratosis, vascular alterations, salivary dysfunction, and persistent inflammatory activation affecting oral soft tissues (122). Clinically, tobacco users may present with a spectrum of oral mucosal alterations, including hyperkeratotic lesions, smoker's palate (nicotinic stomatitis), smoker's melanosis, and leukoplakic changes (122, 123). These alterations commonly coexist with microbial dysbiosis and impaired epithelial barrier integrity, further increasing tissue vulnerability and promoting persistent mucosal instability (82).

Environmental and host-related factors have been investigated as potential modifiers of several immune-mediated and inflammatory oral mucosal disorders. In oral lichen planus, immune dysregulation, oxidative stress, and alterations in local tissue homeostasis have been implicated in disease pathobiology (124). Environmental and systemic factors have also been investigated in recurrent aphthous stomatitis, although their individual contributions remain incompletely defined (125). Exposure to selected dental materials and medications has been implicated in the development of oral lichenoid reactions, particularly in susceptible individuals (126, 127). Although definitive causal relationships remain difficult to establish, available evidence supports the concept that chronic environmental irritation may contribute to the persistence and amplification of mucosal inflammatory activity in susceptible individuals.

Another clinically relevant aspect concerns the influence of environmental exposure on mucosal healing dynamics. Physiological oral mucosal healing requires coordinated epithelial migration, extracellular matrix remodeling, vascular responses, and inflammatory resolution (78). Environmental and other exogenous factors may additionally disrupt epithelial barrier homeostasis, potentially increasing tissue susceptibility to persistent injury (128). Chronic exposure to tobacco smoke and other environmental stressors has been associated with biological alterations involving vascular function, fibroblast activity, and inflammatory signaling (5). These mechanisms may adversely affect mucosal repair; however, the extent of direct clinical evidence for delayed healing, mucosal fragility, ulceration, or secondary infection varies according to the specific exposure considered.

Occupational exposure represents an additional source of chronic mucosal injury (129). Long-term contact with metals, solvents, acids, pesticides, cement particles, and industrial aerosols has been associated with mucosal irritation, burning symptoms, epithelial discoloration, ulcerative lesions, and chronic inflammatory reactions related to repeated chemical exposure (130, 131). Although these manifestations are frequently nonspecific, a detailed occupational history may provide valuable diagnostic information in patients presenting with persistent or otherwise unexplained oral mucosal lesions.

Beyond direct epithelial injury, salivary dysfunction may further compromise mucosal homeostasis (132). Reduced salivary flow or altered salivary composition impair lubrication, antimicrobial defense, buffering capacity, and epithelial protection, thereby increasing susceptibility to irritation and chronic inflammation (133). This mechanism may be particularly relevant in smokers, medically compromised individuals, and subjects exposed to dehydration, medications, or prolonged environmental stress (134, 135). Under these conditions, salivary alterations may exacerbate both the severity and persistence of inflammatory mucosal disease.

#### 3.2.2 Oral potentially malignant disorders, field cancerization, and environmental carcinogenesis

Oral potentially malignant disorders (OPMDs) represent one of the most clinically significant manifestations associated with chronic environmental and lifestyle-related exposures (136). Rather than constituting isolated epithelial abnormalities, these lesions are increasingly recognized as the clinical expression of progressive molecular destabilization affecting chronically injured mucosal fields. Environmental and lifestyle-related exposures may contribute to altered epithelial homeostasis and barrier integrity, although their biological effects and clinical relevance vary considerably according to the exposure considered (137). Tobacco use, alcohol consumption, and ultraviolet radiation represent comparatively well-characterized carcinogenic determinants, whereas evidence concerning several other environmental exposures remains more heterogeneous.

Oral leukoplakia represents the prototypical example of an exposure-associated potentially malignant disorder within the oral cavity. Although clinically defined as a predominantly white plaque that cannot be otherwise classified, its biological behavior remains highly heterogeneous (138). Histopathological findings range from simple hyperkeratosis to severe epithelial dysplasia, reflecting different degrees of molecular instability and malignant potential. Tobacco exposure remains one of the major established risk factors for oral leukoplakia, although a proportion of lesions also occurs in individuals without recognized tobacco exposure (139).

Field cancerization has become a central concept in contemporary oral carcinogenesis. According to this model, sustained carcinogenic and inflammatory injury induces diffuse molecular alterations throughout clinically normal-appearing mucosa, generating multiple epithelial areas with increased susceptibility to dysplasia and malignant transformation (140). Consequently, clinically visible lesions may represent only a limited portion of a much broader biologically altered mucosal field. This concept provides a biological explanation for the occurrence of multifocal lesions, local recurrence following surgical excision, and the development of second primary tumors within previously exposed mucosal regions (141).

Tobacco-derived carcinogens play a pivotal role in this process. Cigarette smoke contains tobacco-specific nitrosamines, polycyclic aromatic hydrocarbons, and other carcinogenic compounds that contribute to cumulative molecular injury in chronically exposed tissues (142). Chronic smoking may also alter salivary microbial composition and local cytokine profiles, further modifying the oral microenvironment (143). Similar biological concerns have emerged regarding smokeless tobacco products, heated tobacco devices, and electronic cigarettes, although their long-term oncogenic implications within oral tissues remain incompletely characterized (17).

Alcohol exposure further enhances epithelial susceptibility through both direct and indirect biological mechanisms. Ethanol and its primary metabolite, acetaldehyde, increase epithelial permeability, facilitate the penetration of carcinogenic compounds, interfere with DNA repair pathways, and amplify oxidative tissue injury (144). In addition, alcohol-associated nutritional deficiencies and salivary dysfunction contribute to impaired epithelial resilience and defective mucosal repair. The combined effects of tobacco and alcohol are well established and substantially increase the risk of epithelial dysplasia and oral squamous cell carcinoma compared with either exposure alone (145).

Persistent inflammatory conditions may also contribute to a tissue microenvironment potentially relevant to OPMD progression. Alterations in immune-cell populations and inflammatory signaling have been described within OPMDs (146). In oral lichen planus, sustained inflammatory activity may coexist with molecular alterations associated with epithelial instability and malignant potential (147). Although the overall risk of malignant transformation remains relatively low, these lesions require careful long-term surveillance, especially when additional carcinogenic risk factors are present.

Among environmental toxicants, heavy-metal exposure has been investigated as a potential risk factor for OPMD progression. Epidemiological evidence from an exposed population has suggested an association between environmental heavy-metal exposure and progression of oral potentially malignant disorders (148). Nevertheless, the observational nature of these data and the possibility of co-exposures and residual confounding preclude firm causal inference.

Another important clinical consideration concerns the diagnostic complexity of OPMDs. Early lesions are frequently asymptomatic and may present as homogeneous leukoplakia, erythroplakia, mixed red-and-white lesions, verrucous lesions, or multifocal mucosal involvement (149, 150). Furthermore, chronic inflammatory and reactive lesions may clinically resemble dysplastic disease, whereas early epithelial transformation may remain histologically subtle (151). Comprehensive exposure assessment, longitudinal clinical follow-up, histopathological evaluation, and individualized risk stratification therefore remain essential components of patient management.

From a surgical perspective, excision of a clinically visible OPMD does not necessarily eliminate molecularly altered mucosa within the surrounding field (140, 141). Consequently, recurrence or the development of additional lesions may occur despite apparently complete local treatment, supporting the need for long-term surveillance and control of modifiable risk factors (140, 141, 152)

#### 3.2.3 Oral squamous cell carcinoma and environment-related carcinogenesis

Oral squamous cell carcinoma (OSCC) develops through the progressive accumulation of molecular, inflammatory, and epithelial alterations induced by persistent carcinogenic stimuli (77). Established environmental and lifestyle-related carcinogenic exposures play an important role in this process, while other environmental determinants remain under investigation (153). The biological pathways involved include genotoxic injury, oxidative stress, immune dysregulation, microbial alterations, and defective epithelial repair. Because the oral mucosa is continuously exposed to inhaled, ingested, and contact-associated toxicants, the oral cavity represents one of the anatomical sites most directly affected by cumulative carcinogenic injury (5).

Tobacco exposure remains the principal environmental determinant associated with OSCC, promoting carcinogenesis through cumulative genotoxic injury, oxidative stress, chronic inflammatory activation, epithelial instability, and impaired immune surveillance (154).

Smokeless tobacco use has also been associated with oral mucosal alterations and increased carcinogenic risk, particularly in populations in which chronic use is prevalent (155). Prolonged mucosal contact with smokeless tobacco products may induce localized keratotic and inflammatory changes and has been associated with epithelial dysplasia (155). The magnitude of carcinogenic risk is influenced by exposure frequency, duration of use, product composition, concomitant alcohol consumption, nutritional status, and individual susceptibility. In parallel, increasing attention has also focused on heated tobacco products and electronic cigarettes. Experimental and clinical evidence suggests that these products may influence oxidative and inflammatory pathways relevant to oral tissue homeostasis (55, 156); however, their long-term oncogenic implications within the oral cavity remain insufficiently characterized.

Alcohol exposure further enhances epithelial susceptibility through increased epithelial permeability, acetaldehyde-mediated toxicity, oxidative injury, and synergistic interactions with tobacco-derived carcinogens (144). Persistent inflammatory activation may contribute to OSCC development and progression by modifying the local tumor microenvironment. Sustained cytokine signaling and other inflammatory mediators may promote cellular proliferation, angiogenesis, extracellular matrix remodeling, and resistance to apoptosis (157).

Accumulating evidence also supports a role for microbiome dysbiosis in oral carcinogenesis through chronic inflammatory activation, epithelial barrier disruption, immune dysregulation, and metabolically altered carcinogenic microenvironments (158). Environmental exposures have also been investigated as potential contributors to oral carcinogenesis, although their relative contribution is difficult to isolate from genetic susceptibility and major lifestyle-related risk factors (159). Available evidence suggests that environmental determinants may contribute to multistage oral carcinogenesis, but the strength and directness of these associations vary considerably according to the exposure considered (159). Current findings therefore support biological plausibility and exposure-associated epithelial vulnerability but do not establish an equivalent level of carcinogenic evidence across these environmental determinants.

Ultraviolet radiation represents a distinct carcinogenic exposure primarily affecting the lip vermilion. Chronic ultraviolet exposure can induce DNA photodamage, oxidative injury, alterations in tumor-suppressor pathways, and local immunological changes that contribute to photocarcinogenesis (105). Actinic cheilitis represents a clinically important potentially malignant disorder associated with cumulative ultraviolet exposure and a risk of progression to lip squamous cell carcinoma (104, 160, 161).

Clinically, OSCC presents with a broad spectrum of manifestations, including non-healing ulceration, induration, exophytic masses, erythroplakia, leukoplakia, mixed red-and-white lesions, and persistent mucosal thickening (162). Early-stage lesions are frequently asymptomatic and may therefore remain undetected for prolonged periods. The lateral border of the tongue, the floor of the mouth, and the soft palate remain among the anatomical sites most susceptible to malignant transformation, particularly in individuals exposed to long-term carcinogenic insults (77).

From a surgical perspective, the concept of field cancerization remains particularly relevant because clinically normal-appearing mucosa surrounding the primary lesion may retain molecular alterations that predispose to local recurrence and second primary tumors. Accordingly, long-term clinical surveillance should be considered an essential component of patient management following treatment of exposure-associated OSCC.

#### 3.2.4 Salivary gland dysfunction and xerostomia-associated alterations

Maintenance of oral tissue homeostasis depends largely on the integrity of salivary gland function. Saliva contributes to lubrication, antimicrobial defense, buffering capacity, mineral homeostasis, mucosal protection, and tissue repair, thereby influencing both hard and soft oral tissues (163). Consequently, alterations in salivary secretion may produce widespread biological effects involving microbial balance, epithelial integrity, mineral regulation, and wound healing (132). Current evidence suggests that selected environmental and lifestyle-related exposures may be associated with alterations in salivary gland function through mechanisms involving vascular dysfunction, inflammatory activation, autonomic imbalance, epithelial injury, and direct effects on secretory tissues (164). The strength of these relationships varies according to the exposure considered.

Xerostomia and hyposalivation are multifactorial conditions commonly associated with aging, medication use, systemic disease, and other alterations affecting salivary gland function (165). Environmental and lifestyle-related exposures have also been investigated as potential modifiers of salivary secretion, although the evidence differs considerably among individual exposures. These factors may alter both salivary flow and biochemical composition, compromising several protective functions of saliva within the oral cavity.

Tobacco exposure is among the most extensively investigated lifestyle-related factors affecting salivary physiology. Chronic smoking has been associated with alterations in salivary secretion and salivary gland function (166). Several studies have also documented modifications in salivary pH, buffering capacity, immunoglobulin concentrations, antioxidant activity, and inflammatory mediators among smokers (167). Clinically, tobacco-associated xerostomia is frequently accompanied by mucosal irritation, microbial dysbiosis, impaired taste perception, increased caries susceptibility, halitosis, and delayed epithelial healing (168).

Alcohol consumption may also affect oral and salivary homeostasis (169). Experimental evidence further indicates that chronic alcohol exposure can interfere with salivary gland regenerative processes (170). However, the clinical magnitude of alcohol-associated salivary dysfunction remains less clearly defined than that of several established systemic and pharmacological causes of xerostomia.

Environmental and occupational exposures may also affect salivary gland homeostasis (5). Heavy metals, industrial solvents, pesticides, cement dust, and airborne particulate matter have all been associated with glandular irritation, altered secretory activity, and epithelial injury involving salivary tissues (171). Occupational exposure has also been associated with subjective oral dryness in selected worker populations. In a cross-sectional study of bone-factory workers, abnormal dry mouth was frequently reported, although the contribution of occupational exposure could not be clearly separated from other relevant factors, including tobacco use (172).

Salivary dysfunction also has important consequences for the oral ecosystem. Reduced salivary flow impairs microbial clearance, buffering capacity, and antimicrobial defense, thereby facilitating biofilm accumulation and microbial dysbiosis (173). Simultaneously, diminished lubrication and epithelial protection increase susceptibility to chronic irritation, ulceration, opportunistic infections, and inflammatory mucosal disorders. These biological alterations may also compromise wound healing and increase postoperative discomfort following oral surgical procedures. In addition to its effects on soft tissues, salivary dysfunction influences the integrity of dental hard tissues. Saliva plays a fundamental role in enamel remineralization and maintenance of mineral equilibrium within the oral environment (174). Hyposalivation therefore increases susceptibility to dental erosion, root caries, cervical demineralization, and accelerated progression of existing dental disease (175). These effects become particularly relevant in individuals simultaneously exposed to acidic dietary habits, tobacco products, gastroesophageal reflux, or persistent dehydration.

Chronic inflammatory and autoimmune disorders affecting salivary glands may further interact with environmental exposures. Persistent glandular inflammation, epithelial injury, and altered secretory homeostasis may aggravate xerostomia-associated symptoms and oral complications, particularly in susceptible individuals presenting with additional systemic or pharmacological risk factors (176). Clinically, salivary dysfunction frequently presents with nonspecific manifestations including oral dryness, burning sensation, dysphagia, taste alteration, mucosal fragility, halitosis, and difficulty wearing removable prostheses. Because symptoms may precede objectively measurable reductions in salivary flow, careful clinical assessment is particularly important in chronically exposed or medically compromised patients. Evaluation of environmental and occupational exposures, systemic diseases, medication use, hydration status, and lifestyle-related risk factors provides valuable information for diagnosis and clinical management.

#### 3.2.5 Peri-implant soft tissue alterations and exposure-associated complications

Long-term implant stability depends largely on the integrity of peri-implant soft tissues, which are essential for biological sealing, microbial regulation, vascular homeostasis, and maintenance of peri-implant tissue health (177). Environmental and lifestyle-related exposures have been investigated for their potential influence on peri-implant tissues through interconnected mechanisms involving inflammatory dysregulation, impaired epithelial maturation, altered immune responses, microbial dysbiosis, and vascular dysfunction (39). Because peri-implant tissues lack the structural organization and vascular supply provided by the periodontal ligament, they appear particularly vulnerable to persistent environmental and inflammatory challenges (69).

Smoking remains one of the most extensively investigated risk factors associated with peri-implant soft tissue complications. Smoking remains one of the most extensively investigated exposure-related risk factors for peri-implant complications. Clinical evidence indicates that smoking adversely affects several peri-implant parameters and may compromise peri-implant tissue health and treatment outcomes (178). Chronic smoking has also been associated with increased inflammatory infiltrates, altered cytokine profiles, defective collagen metabolism, and delayed peri-implant mucosal maturation. Clinically, smokers consistently exhibit greater plaque accumulation, peri-implant mucositis, persistent mucosal inflammation, and an increased risk of peri-implantitis progression (179).

Microbial dysbiosis represents another key mechanism involved in exposure-associated peri-implant disease. Tobacco use and chronic inflammatory conditions modify peri-implant biofilm composition, favoring colonization by pathogenic anaerobic species associated with tissue destruction and peri-implant bone loss (39). Dysbiotic microbial communities further sustain chronic inflammatory activity and impair peri-implant mucosal stability. Electronic cigarette use has also been associated with adverse changes in clinical, radiographic, and immunological peri-implant parameters, including increased probing depth, marginal bone loss, and peri-implant IL-1β levels (180). However, the available evidence remains limited, and the long-term implications of vaping for peri-implant disease require further investigation.

Environmental and occupational pollutants have been associated with oxidative, inflammatory, and epithelial alterations in different biological systems (5). Whether these effects translate into clinically relevant alterations of peri-implant soft tissues remains largely uncertain. Direct clinical evidence specifically linking air pollution, heavy metals, or other occupational toxicants to peri-implant mucosal complications is currently limited.

Peri-implant mucosal healing depends on coordinated epithelial migration, fibroblast proliferation, angiogenesis, extracellular matrix organization, and immune regulation. Persistent environmental stress may interfere with these biological processes by altering vascular homeostasis, sustaining low-grade inflammatory activity, and reducing reparative cellular capacity (5). These mechanisms provide a biological basis for potential alterations in peri-implant mucosal maturation, soft tissue sealing, and postoperative healing; however, direct clinical evidence remains limited for several environmental exposures (118).

A related aspect concerns the interaction between implant biomaterials and the local immune-inflammatory microenvironment, since material properties may influence biological responses relevant to peri-implant tissue homeostasis (181). Implant surface characteristics can influence both osseointegration and biofilm formation, highlighting the importance of biomaterial–host–microbial interactions in peri-implant tissue homeostasis (182). In susceptible individuals, these biological interactions have been associated with progressive peri-implant tissue instability and an increased risk of peri-implant disease progression.

Salivary dysfunction may further exacerbate peri-implant soft tissue complications. Salivary dysfunction may further influence peri-implant health. Clinical evidence suggests that xerostomia may be associated with reduced implant success and alterations in peri-implant bone stability, although the available evidence remains limited (183). These effects may become particularly relevant in smokers, medically compromised individuals, and patients presenting with chronic xerostomia. Clinically, peri-implant inflammatory disease may present with mucosal inflammation and bleeding on probing, with progressive tissue destruction characterizing more advanced disease (184). Regular peri-implant monitoring therefore remains essential, particularly in patients presenting with established modifiable risk factors such as smoking.

### 3.3 Environment-associated diseases of hard oral tissues

Mineralized oral tissues are continuously influenced by chemical, microbial, mechanical, dietary, systemic, and environmental determinants, while their structural integrity depends on interactions among salivary protection, microbial ecology, mineral availability, and tissue-specific biological responses (13, 185). These determinants have been investigated in relation to dental caries, erosive tooth wear, cervical hard tissue alterations, developmental defects, and changes in alveolar bone homeostasis. Tobacco exposure, for example, has been associated with increased caries prevalence (186), whereas repeated exposure to dietary, intrinsic, or occupational acids is well established in the pathogenesis of erosive tooth wear (187, 188). Gingival recession, periodontal alterations, acidic exposure, and other environmental or behavioral determinants have also been investigated in relation to dentin hypersensitivity and cervical hard tissue lesions (189, 190).

Environmental and lifestyle-related determinants have additionally been investigated in relation to alveolar bone and dental mineralization. Among lifestyle-related exposures, smoking has been associated with periodontal inflammation and alveolar bone resorption (191). Other systemic, nutritional, and environmental determinants may also influence bone remodeling, although the strength of evidence varies according to the specific exposure and clinical outcome. Heavy metals and industrial toxicants have been investigated for their potential effects on bone and mineral homeostasis (5). At the local oral level, mineral availability may also be influenced by restorative biomaterials. Experimental evidence indicates that contemporary restorative materials can release calcium, phosphate, and fluoride ions, although the magnitude and kinetics of release vary substantially according to material composition (192). However, the direct clinical relevance of these findings varies substantially according to the exposure and outcome considered. Salivary dysfunction represents a further cross-cutting determinant, as reduced protective capacity may favor prolonged acid exposure, microbial persistence, demineralization, and impaired remineralization (13, 193).

Overall, the evidentiary maturity of environment-related hard tissue alterations is heterogeneous. Established relationships, such as those involving acidic exposure and erosive tooth wear, coexist with predominantly observational or mechanistically supported relationships involving environmental pollutants, heavy metals, developmental alterations, and regenerative outcomes. Clinically reported manifestations encompass carious lesions, erosive wear, enamel alterations, cervical defects, hypersensitivity, root-surface demineralization, and alveolar bone changes (194).

#### 3.3.1 Dental caries and exposure-associated microenvironmental alterations

Development of dental caries reflects progressive disruption of the ecological equilibrium regulating mineralized tooth surfaces. Although traditionally recognized as a biofilm-mediated disease driven by bacterial metabolism of fermentable carbohydrates, caries progression results from complex interactions involving microbial activity, dietary habits, salivary protection, mineral homeostasis, behavioral determinants, and environmental exposures. Current evidence indicates that caries susceptibility is associated with multiple environmental, behavioral, and lifestyle-related determinants acting through microbial ecology, salivary function, dietary exposure, enamel resistance, and oral biofilm dynamics (195). The relative contribution and strength of evidence differ among individual determinants.

The oral microenvironment plays a fundamental role in preserving mineralized tissue integrity (185). Physiological homeostasis depends on the continuous balance between demineralization and remineralization regulated by salivary buffering capacity, calcium and phosphate availability, fluoride exposure, microbial composition, dietary habits, and oral hygiene practices. Persistent disruption of this ecological equilibrium favors prolonged acidification of dental biofilms, thereby promoting mineral loss and progressive destruction of enamel and dentin (185).

Tobacco exposure has been consistently associated with increased caries prevalence and severity (186). Chronic smoking alters salivary secretion, reduces buffering capacity, impairs antimicrobial defense, and promotes accumulation of cariogenic biofilms (196). Several studies have also documented smoking-associated shifts in the oral microbiome characterized by increased abundance of acidogenic and aciduric bacterial species (197). Together, these alterations provide plausible pathways through which tobacco exposure may contribute to increased caries susceptibility, particularly in individuals with inadequate oral hygiene or reduced salivary protection.

Dietary habits represent another major determinant of caries susceptibility. Frequent consumption of fermentable carbohydrates and acidic beverages promotes prolonged reductions in oral pH and sustained cariogenic biofilm activity (198). Nutritional deficiencies and inadequate mineral intake may further reduce enamel resistance and compromise remineralization capacity. Socioeconomic factors also influence disease burden by limiting access to preventive care, promoting unhealthy dietary patterns, and increasing exposure to lifestyle-related risk factors such as tobacco and alcohol use (199).

Reduced salivary protection further amplifies caries risk by impairing buffering activity, microbial clearance, mineral transport, and antimicrobial defense. Decreased salivary flow facilitates prolonged retention of dietary acids and bacterial metabolites on tooth surfaces, thereby accelerating demineralization and progression of existing lesions (200). These mechanisms are particularly relevant in smokers, medically compromised patients, older adults, and individuals presenting with dehydration or medication-associated xerostomia.

Environmental and occupational exposures may additionally contribute to ecological alterations associated with caries susceptibility. Airborne pollutants, acidic industrial environments, particulate matter, heavy metals, and occupational chemical agents have been associated with enamel surface alterations, mineral destabilization, salivary dysfunction, and increased susceptibility to cervical demineralization (5, 201). However, the available evidence is heterogeneous, and interpretation is complicated by differences in exposure assessment and by potential confounding from socioeconomic, dietary, occupational, and oral hygiene-related factors. These findings therefore support an association rather than a direct causal relationship between such environmental exposures and caries susceptibility.

Microbial dysbiosis represents another important component of exposure-associated caries development. Alterations in the oral ecosystem may favor proliferation of acidogenic microbial communities capable of maintaining a persistently acidic biofilm environment and sustaining mineral dissolution (185). Tobacco exposure has been associated with alterations in oral microbial diversity and composition, providing an additional pathway through which smoking may influence oral ecological homeostasis (82).

Clinically, caries lesions may present across a continuum ranging from initial non-cavitated enamel changes, including white-spot lesions, to progressively more extensive cavitated lesions involving enamel and dentin (202). Root caries are particularly common in individuals presenting with gingival recession, hyposalivation, periodontal disease, or chronic tobacco exposure. Because early lesions frequently remain asymptomatic, comprehensive assessment of dietary habits, salivary function, environmental exposures, and behavioral risk factors remains essential for accurate risk assessment and timely preventive intervention.

#### 3.3.2 Dental erosion and chemically induced mineralized tissue loss

Dental erosion represents a chronic chemical process characterized by progressive dissolution of enamel and dentin caused by exposure to non-bacterial acids. Unlike carious disease, erosive tooth wear occurs independently of microbial acid production and primarily reflects imbalance between acidic challenge and protective mechanisms regulating mineralized tissue stability (187). Erosive tooth wear is associated with a range of dietary, intrinsic, occupational, and behavioral acid exposures, although the strength of evidence differs among individual determinants (203). Enamel resistance to acidic dissolution relies on effective buffering activity, remineralization dynamics, salivary clearance mechanisms, and acquired pellicle protection. Repeated acidic challenges promote progressive mineral loss from enamel, with the magnitude of surface alteration influenced by the conditions surrounding acid exposure (204). Because enamel exhibits limited regenerative capacity, prolonged erosive injury may produce cumulative and irreversible structural damage.

Dietary acids represent one of the principal factors associated with dental erosion. Frequent consumption of carbonated beverages, citrus products, sports drinks, energy drinks, and acidic dietary supplements has been consistently associated with increased erosive tooth wear (205). Recurrent reductions in oral pH facilitate hydroxyapatite dissolution and progressive enamel softening, particularly when acidic exposure occurs over prolonged periods or in the presence of reduced salivary protection (186). Intrinsic acidic exposure also plays a major role in erosive mineralized tissue loss. Gastroesophageal reflux disease, recurrent vomiting, eating disorders, alcoholism-associated emesis, and chronic gastric acid regurgitation expose oral tissues to highly acidic conditions capable of inducing extensive enamel dissolution (188). Palatal surfaces of maxillary teeth and occlusal surfaces frequently exhibit characteristic erosive patterns in individuals presenting with chronic intrinsic acid exposure (206).

Occupational acidic conditions represent another relevant determinant of dental erosion. Workers chronically exposed to industrial acidic vapors, battery manufacturing environments, chemical processing agents, or corrosive industrial aerosols demonstrate increased prevalence of erosive enamel alterations and mineralized tissue defects (207). Occupational exposure to wine has also been associated with dental erosion and dentinal sensitivity among professional wine tasters (209). Exposure to acidic swimming-pool environments has additionally been investigated as a potential contributor to erosive tooth wear, although part of the available evidence is experimental (208).

Salivary protection plays a critical role in limiting erosive tissue injury. Saliva contributes to buffering acidic compounds, promoting remineralization, lubricating tooth surfaces, and facilitating clearance of erosive substances from the oral cavity (210). Reduced salivary flow, impaired buffering activity, dehydration, smoking-associated salivary alterations, and medication-induced xerostomia significantly increase susceptibility to erosive mineral loss and accelerate progression of existing lesions (211).

Mechanical and behavioral factors may additionally interact with erosive processes and amplify structural tissue damage. Tooth brushing performed immediately after acidic exposure, parafunctional activity, abrasive oral hygiene practices, and occlusal stress contribute to removal of softened enamel surfaces and progression of dentin exposure (212). In many individuals, erosive tooth wear therefore reflects combined interaction among chemical, mechanical, and behavioral determinants.

Environmental airborne contaminants and industrial pollutants have also been associated with alterations affecting enamel surface integrity and mineral stability (213). Exposure to acidic particulate matter, industrial chemicals, combustion-derived pollutants, and contaminated aerosols has been linked to increased enamel roughness, mineral dissolution, and structural surface alterations (5, 214). Although associations with enamel surface alterations and erosive wear have been reported, the available evidence for airborne contaminants and non-acid-specific industrial pollutants remains heterogeneous and largely observational (5, 214).

Clinically, dental erosion may present through enamel smoothening, loss of surface texture, incisal translucency, cupping defects, dentin exposure, cervical lesions, hypersensitivity, and progressive reduction of dental structure (215). Because early erosive lesions may initially remain asymptomatic, careful evaluation of dietary habits, occupational exposure, salivary status, systemic conditions, and behavioral risk factors remains essential during clinical assessment. Despite substantial advances in understanding erosive mechanisms, important limitations remain. Many studies investigating exposure-associated erosive wear are observational and complicated by overlap among dietary habits, salivary alterations, behavioral factors, systemic disease, smoking, and occupational exposure. Significant heterogeneity also persists regarding diagnostic indices, lesion classification, and quantification of erosive progression. Overall, the evidence most consistently supports a role for repeated acidic exposure and impaired salivary protection in erosive tooth wear, whereas the contribution of broader environmental and occupational exposures depends on the specific agent and remains less consistently characterized.

#### 3.3.3 Alveolar bone loss and environment-associated bone remodeling alterations

Integrity of alveolar bone depends on tightly regulated remodeling processes involving the coordinated balance between osteoblastic bone formation and osteoclastic resorption (216). These biological mechanisms are influenced by vascular supply, inflammatory mediators, mechanical loading, hormonal regulation, nutritional status, and microbial homeostasis within periodontal and peri-implant tissues. Several environmental and lifestyle-related factors have been investigated in relation to alveolar bone remodeling (217). Their effects appear to involve inflammatory, vascular, metabolic, and cellular pathways; however, the strength of direct clinical evidence varies substantially among individual exposures and outcomes.

Smoking remains one of the principal environmental factors associated with alveolar bone loss. Tobacco-derived compounds impair vascular perfusion, reduce oxygen delivery, interfere with osteoblastic differentiation, and alter collagen metabolism within periodontal tissues (191). Chronic tobacco exposure has also been associated with increased osteoclastic activity, impaired angiogenesis, altered cytokine expression, and reduced regenerative potential (110). These mechanisms are consistent with the established clinical association between smoking and periodontal destruction, implant-related complications, and impaired healing.

Periodontal inflammation represents another major determinant of environmentally influenced alveolar bone destruction (111). Persistent microbial dysbiosis and chronic inflammatory activity stimulate the release of cytokines, prostaglandins, matrix metalloproteinases, and osteoclast-activating mediators that promote progressive bone resorption (39). Tobacco exposure, systemic inflammatory disorders, impaired metabolic control, and inadequate oral hygiene further amplify these tissue-destructive pathways, accelerating periodontal attachment loss and alveolar bone destruction (44).

Systemic metabolic disorders also exert a significant influence on alveolar bone remodeling. Diabetes mellitus, osteoporosis, nutritional deficiencies, hormonal imbalance, and chronic inflammatory diseases have all been associated with altered bone metabolism and impaired regenerative responses within oral tissues (218, 219). Hyperglycemia-related oxidative stress, microvascular dysfunction, and sustained inflammatory signaling reduce osteoblastic activity and delay periodontal and peri-implant healing. Similar alterations have also been described in individuals presenting with disorders of skeletal metabolism or prolonged deficiencies affecting calcium and vitamin D homeostasis (220).

Environmental toxicants and heavy metals have also been investigated in relation to bone remodeling (5). Experimental and toxicological evidence indicates that cadmium, lead, mercury, and selected industrial contaminants may interfere with osteoblastic differentiation, calcium metabolism, mineral deposition, and extracellular bone matrix organization (221). Associations between environmental exposure and alterations in bone mineral density have also been reported (222). However, direct evidence linking these exposures to alveolar bone loss, impaired osseous repair, or clinically relevant periodontal and peri-implant regenerative outcomes remains limited.

Occupational exposures have also been investigated in relation to periodontal and alveolar bone alterations, particularly in populations chronically exposed to industrial chemicals, airborne particulate matter, and other irritants (86). Although associations with periodontal destruction and inflammatory alterations have been reported (223), the available evidence remains heterogeneous, and the independent contribution of individual occupational exposures is difficult to establish because of mixed exposures and potential behavioral, socioeconomic, and systemic confounding.

Nutritional and behavioral determinants further influence alveolar bone stability. Nutritional and behavioral determinants may further influence alveolar bone stability. Inadequate protein intake, vitamin deficiencies, excessive alcohol consumption, physical inactivity, and chronic psychological stress have been investigated in relation to bone turnover and regenerative capacity (224). At the same time, poor oral hygiene and delayed periodontal treatment contribute to persistence of inflammatory microenvironments that favor progressive alveolar bone resorption (225).

Microbial dysbiosis also contributes to disruption of alveolar bone remodeling by sustaining osteoclast-activating inflammatory pathways within periodontal and peri-implant tissues (35). Reduced salivary protection and impaired host defense facilitate microbial persistence, maintaining chronic inflammatory conditions that promote connective tissue degradation and progressive loss of tooth- and implant-supporting structures (44).

Because early remodeling abnormalities frequently remain clinically silent, radiographic evaluation combined with comprehensive assessment of systemic diseases, environmental exposures, and lifestyle-related risk factors remains fundamental for periodontal and peri-implant maintenance.

#### 3.3.4 Developmental defects and environment-associated alterations of dental mineralization

Normal odontogenesis requires tightly coordinated ameloblastic and odontoblastic activity throughout enamel and dentin formation (226). These developmental processes depend on balanced mineral metabolism, adequate nutritional support, hormonal regulation, and stable systemic and environmental conditions during critical stages of tissue maturation. Disruption of these mechanisms during odontogenesis may result in structural defects affecting enamel and dentin, characterized by altered mineralization, defective matrix formation, and increased susceptibility to long-term dental deterioration (227). Developmental defects of dental mineralization have been associated with a range of nutritional, systemic, toxicological, and environmental factors, although their relative contribution and evidentiary support vary considerably among individual exposures.

Enamel formation is particularly sensitive to systemic and environmental disturbances because ameloblasts exhibit high metabolic activity and lack regenerative capacity (228). Alterations occurring during the secretory or maturation phases of amelogenesis may produce quantitative defects, such as enamel hypoplasia, or qualitative defects, including hypomineralization and altered enamel translucency. These structural abnormalities compromise enamel resistance, facilitate plaque accumulation, and increase susceptibility to dental caries, dentin hypersensitivity, and erosive tooth wear (229).

Nutritional deficiencies remain among the principal determinants associated with developmental mineralization defects. Inadequate intake of calcium, phosphate, vitamin D, vitamin A, and protein during odontogenesis interferes with mineral deposition and enamel maturation (230). Early-life nutritional imbalance and prolonged malnutrition have consistently been associated with enamel hypoplasia, delayed tooth eruption, and defective mineralized tissue development, particularly in socioeconomically disadvantaged populations (231).

Environmental toxicants and heavy metals have also been investigated in relation to developmental dental mineralization (5). Experimental and observational findings suggest that exposure to lead, cadmium, mercury, arsenic, and selected industrial contaminants may interfere with ameloblastic activity, enamel matrix organization, crystal maturation, and calcium-phosphate homeostasis during odontogenesis (232). Exposure during critical developmental stages has been associated with enamel hypomineralization, structural opacities, reduced mineral density, and increased long-term susceptibility to dental deterioration (233). Similar alterations have also been described in populations exposed to industrial contamination, polluted water sources, and airborne environmental pollutants (234).

Excessive fluoride exposure during odontogenesis represents another important environmental determinant affecting enamel mineralization. Dental fluorosis results from disruption of enamel maturation induced by prolonged fluoride overexposure during tooth development (235). Clinically, fluorotic alterations range from mild enamel opacities to severe hypomineralization associated with surface porosity, discoloration, and structural fragility. The severity of these defects is influenced by the duration and intensity of fluoride exposure, nutritional status, renal function, and individual susceptibility during critical stages of enamel development (236).

Systemic diseases and metabolic disorders have also been associated with developmental alterations affecting mineralized dental tissues. Childhood infections, chronic inflammatory diseases, endocrine disorders, renal disease, and metabolic disturbances have all been associated with impaired enamel formation and defective dentin mineralization (237). Likewise, perinatal complications, low birth weight, hypoxia, and prolonged systemic illness during early childhood have been associated with developmental enamel defects involving the permanent dentition (238).

Prenatal and early-life environmental exposures have been investigated in relation to subsequent alterations in dental development and mineralization (239). Maternal smoking, alcohol consumption, environmental pollution, nutritional deficiencies, and toxic exposures during pregnancy have been associated with disturbances affecting fetal dental development (240). However, these relationships are predominantly observational and may be influenced by coexisting maternal, socioeconomic, nutritional, and systemic factors. Their independent contribution to enamel quality, tooth eruption patterns, and long-term structural resistance therefore remains difficult to establish.

Developmental mineralization defects also modify the oral ecosystem and increase long-term susceptibility to oral disease. Rough enamel surfaces, structural porosity, and defective mineral organization facilitate biofilm accumulation and promote progression of dental caries, erosive tooth wear, and dentin hypersensitivity (241). In more severe cases, extensive structural defects may compromise esthetics, masticatory function, and long-term preservation of the affected dentition (242). Because many of these defects originate during early childhood and may remain unrecognized for prolonged periods, careful evaluation of prenatal history, systemic diseases, nutritional status, fluoride exposure, and environmental risk factors remains essential during clinical assessment. Overall, current evidence supports a multifactorial interpretation of developmental defects of dental mineralization, involving interactions among environmental exposures, nutritional status, systemic health, and individual susceptibility during odontogenesis. The relative contribution of individual environmental exposures remains difficult to isolate, particularly when evidence is derived predominantly from observational studies.

#### 3.3.5 Root surface alterations and environment-associated non-carious cervical lesions

Exposure of cervical root surfaces creates a biologically vulnerable interface between mineralized tissues and the oral environment (243). Unlike enamel-covered coronal structures, exposed root surfaces exhibit reduced mineralization and increased susceptibility to demineralization, abrasion, hypersensitivity, and structural degradation (244). Multiple local, behavioral, and environmental determinants have been associated with the development and progression of cervical mineralized tissue defects through interconnected mechanisms involving gingival recession, salivary alterations, acidic dissolution, periodontal destruction, and impaired tissue integrity (245).

Root surface exposure frequently results from gingival recession associated with periodontal disease, aging, traumatic oral hygiene practices, smoking, or chronic inflammatory conditions (246). Loss of periodontal soft tissue coverage facilitates direct contact between cervical dentin and the oral environment, thereby increasing susceptibility to mechanical abrasion, erosive dissolution, microbial colonization, and hypersensitivity (247). Consequently, cervical tissue alterations often reflect the combined effects of periodontal breakdown, environmental exposures, and behavioral factors rather than a single pathogenic mechanism.

Mechanical abrasion represents one of the principal contributors to non-carious cervical lesions (NCCLs). Aggressive tooth brushing, excessive brushing frequency, abrasive dentifrices, and inappropriate oral hygiene techniques progressively contribute to the loss of cervical mineralized tissues (248). These alterations become even more pronounced in the presence of pre-existing erosive enamel softening or gingival recession exposing root dentin surfaces.

Smoking and chronic periodontal inflammation may further increase cervical tissue vulnerability. Tobacco exposure has been associated with periodontal alterations and changes affecting dental hard tissues (249), while gingival recession and periodontal breakdown may increase exposure of cervical dentin and root surfaces. Persistent periodontal inflammation may therefore indirectly increase susceptibility to cervical structural deterioration and root caries (250).

Salivary protection plays a major role in preserving cervical mineralized tissue integrity. Saliva contributes to lubrication, buffering activity, antimicrobial defense, and remineralization of exposed dentin surfaces (174). Reduced salivary flow, medication-associated xerostomia, dehydration, smoking-associated salivary alterations, and systemic diseases compromise these protective mechanisms and increase susceptibility to cervical demineralization and dentin hypersensitivity (211).

Occupational exposure to acidic environments may represent an additional determinant of cervical hard tissue alterations. An association between occupational exposure to acid mists and the prevalence of non-carious cervical lesions has been reported (251). However, direct clinical evidence concerning broader categories of airborne pollutants, industrial particulate matter, and other environmental contaminants remains limited. Accordingly, the contribution of occupational or environmental exposures to cervical tissue deterioration should be interpreted in relation to the specific agent and exposure context rather than as a uniform effect of environmental burden.

Microbial dysbiosis further contributes to root surface pathology following gingival recession and exposure of cervical dentin (252). Dysbiotic biofilms facilitate persistence of acidic microenvironments and promote root caries progression, particularly in older adults, smokers, and individuals presenting with impaired salivary defense mechanisms (253). Root surface lesions often progress rapidly because exposed dentin is considerably less resistant to demineralization than enamel-covered coronal structures.

### 3.4 Salivary dysfunction as a mediator of oral exposome-associated tissue alterations

Saliva represents a major biological interface between environmental exposures and oral tissues because of its roles in lubrication, buffering, antimicrobial defense, mineral homeostasis, and mucosal protection (38). Within the oral exposome framework, salivary dysfunction may therefore be interpreted as a cross-tissue modifier of the local response to environmental and host-related determinants rather than as an isolated pathological process (10). This perspective is particularly relevant because alterations in salivary flow or composition can simultaneously affect microbial ecology, mineral balance, and mucosal integrity.

A range of environmental, behavioral, and systemic factors has been investigated in relation to salivary gland physiology and the protective functions of saliva (5). Tobacco exposure, dehydration, medication use, chronic systemic diseases, nutritional imbalance, and selected occupational conditions have been associated with alterations in salivary secretion, buffering capacity, or antimicrobial activity (254). Aging and polypharmacy may further contribute to salivary impairment, particularly in medically compromised individuals (255). However, the strength and directness of these relationships vary according to the determinant considered.

Clinically, reduced salivary protection may modify susceptibility to both hard- and soft-tissue disorders by affecting microbial clearance, acid neutralization, remineralization, lubrication, and mucosal defense (32, 173). Salivary dysfunction may therefore provide a biologically relevant link among several exposure-associated oral outcomes discussed in the preceding sections without implying a uniform causal pathway across them. Assessment of salivary symptoms and relevant systemic, pharmacological, behavioral, and environmental determinants may provide useful complementary information during oral risk assessment (13).

The principal exposure-associated biological alterations affecting oral tissues and their potential clinical implications within the framework of the oral exposome are summarized in Table 2.

**Table 2:** Integrated overview of the principal biological systems affected by the oral exposome, their exposure-associated alterations, underlying biological consequences, and potential clinical implications.

Target tissue/biological system	Principal exposure-associated biological alterations	Main biological consequences	Potential clinical implications
Oral microbiome	Ecological disruption, reduced microbial diversity, dysbiotic biofilm development, altered microbial metabolism	Loss of microbial homeostasis, persistent inflammation, altered host–microbiome interactions	Increased susceptibility to dental caries, periodontitis, peri-implant disease, and opportunistic infections; potential contribution to impaired tissue healing
Salivary system	Reduced salivary flow, impaired buffering capacity, altered antimicrobial activity, ecological imbalance	Defective microbial clearance, impaired remineralization, mucosal dryness, reduced tissue protection	Xerostomia, increased caries risk, mucosal discomfort, dysbiosis, impaired healing
Oral epithelium and mucosal barrier	Barrier dysfunction, oxidative stress, chronic irritation, impaired epithelial renewal, genotoxic injury	Reduced epithelial integrity, increased permeability, chronic inflammation, impaired repair	Chronic mucosal disorders, opportunistic infections, oral potentially malignant disorders (OPMDs), oral squamous cell carcinoma (OSCC)
Periodontal tissues	Oxidative stress, chronic inflammatory activation, dysbiotic biofilms, immune dysregulation	Connective tissue degradation, collagen breakdown, osteoclast activation, periodontal attachment loss	Periodontitis, progressive periodontal destruction, tooth-support loss
Peri-implant tissues	Dysbiosis, persistent inflammatory response, endothelial dysfunction, impaired soft tissue integration	Altered peri-implant inflammatory homeostasis, connective tissue instability, inflammatory bone resorption, and potential impairment of tissue integration	Peri-implant mucositis and peri-implantitis; potential impairment of peri-implant tissue stability and osseointegration depending on the specific exposure
Dental hard tissues	Acidic microenvironment, altered mineral homeostasis, impaired remineralization, salivary dysfunction	Enamel and dentin demineralization, structural weakening, defective mineral recovery	Dental caries, erosive tooth wear, non-carious cervical lesions (NCCLs), dentin hypersensitivity
Alveolar bone and regenerative tissues	Chronic inflammatory burden, altered osteoblast–osteoclast balance, endothelial dysfunction, impaired vascular support	Altered bone remodeling and potential impairment of regenerative and mineralized tissue repair processes	Alveolar bone alterations; potential effects on osseous healing, regenerative outcomes, and graft integration
Healing and reparative response	Persistent oxidative stress, fibroblast dysfunction, impaired angiogenesis, extracellular matrix dysregulation	Altered tissue maturation and biological processes involved in wound healing and regeneration	Potential delayed post-operative healing and compromised regenerative outcomes, with clinical relevance varying according to the specific exposure
Oral epithelial carcinogenic pathway	Chronic genotoxic exposure, oxidative DNA damage, epigenetic dysregulation, persistent inflammatory signaling	Genomic instability, impaired apoptosis, abnormal epithelial proliferation	Increased susceptibility to epithelial dysplasia, OPMDs, and OSCC

The biological consequences and potential clinical implications summarized in this table represent an integrated overview across different environmental and host-related determinants and should not be interpreted as being supported by equivalent levels of evidence for all exposures. The strength and directness of the evidence vary according to the specific exposure–outcome relationship. A qualitative appraisal of evidentiary maturity for the principal relationships is provided in Table 3.

Taken together, the evidence synthesized across the different oral tissues and biological systems reveals substantial differences in evidentiary maturity among individual exposure–outcome relationships. To make these differences explicit and to distinguish well-supported relationships from those for which the available evidence remains predominantly associative, emerging, or hypothesis-generating, the principal exposure–outcome relationships identified throughout this review were qualitatively appraised according to the predominant source and directness of the available evidence, the extent and consistency of human data, and the contribution of experimental or mechanistic findings. The resulting evidence-strength matrix is presented in Table 3.

**Table 3:** Qualitative evidence-strength matrix of the principal environmental exposure–oral outcome relationships identified in the current literature.

Environmental exposure	Principal oral outcome(s)	Predominant evidence base	Main findings in human studies	Main limitations and uncertainties	Evidence interpretation	Representative references
Conventional tobacco smoking	Periodontal inflammation, attachment loss, and periodontal tissue destruction	Clinical and epidemiological studies supported by extensive mechanistic evidence	Substantial and generally consistent	Heterogeneity in exposure intensity and duration; residual confounding by behavioral and systemic factors	Established	(15, 53, 54)
Conventional tobacco smoking	Impaired wound healing, osseointegration, and implant-related complications	Clinical evidence supported by vascular, inflammatory, and cellular studies	Substantial, particularly for healing and implant-related outcomes	Heterogeneity in smoking exposure, implant characteristics, patient susceptibility, and outcome definitions	Established	(15, 111, 112)
Conventional tobacco smoking	OPMDs, epithelial dysplasia, and OSCC	Epidemiological and clinical evidence supported by extensive molecular and carcinogenic evidence	Substantial and consistent	Interaction with alcohol and other carcinogenic exposures may complicate attribution of individual effects	Established	(15, 97, 145, 154)
Electronic cigarettes and heated tobacco products	Periodontal, mucosal, salivary, and oral microbiome alterations	Observational human studies and evidence syntheses supported by experimental and translational studies	Available but comparatively limited, particularly for long-term clinical outcomes	Product heterogeneity, evolving devices and formulations, dual use, relatively short exposure histories, and limited longitudinal evidence	Emerging	(16, 17, 55, 123, 168, 180)
Alcohol exposure	Oral mucosal injury, epithelial dysplasia, OPMDs, and oral carcinogenesis	Clinical and epidemiological evidence supported by established toxicological mechanisms	Substantial, particularly for carcinogenic outcomes	Frequent co-exposure to tobacco; heterogeneity in dose, duration, and drinking patterns	Established	(145, 169)
Air pollution and particulate matter	Periodontal inflammation and periodontal disease	Predominantly observational and epidemiological evidence supported by mechanistic studies	Increasing but predominantly observational	Exposure misclassification, geographic variability, residual confounding, and limited longitudinal oral-specific evidence	Supported but predominantly associative	(5, 14, 19, 56)
Air pollution and particulate matter	Alveolar bone alterations, impaired wound healing, and reduced regenerative potential	Predominantly experimental and mechanistic evidence with indirect human support	Limited for oral regenerative outcomes	Direct clinical evidence involving oral regeneration and implant-related healing remains scarce	Hypothesis-generating	(22, 113)
Heavy metals and industrial toxicants	Oral mucosal alterations, epithelial/genotoxic injury, and OPMD progression	Observational human evidence combined with toxicological and mechanistic studies	Available for selected metals and outcomes	Considerable heterogeneity among metals, exposure routes, doses, and co-exposures; potential occupational and lifestyle confounding	Supported but predominantly associative	(5, 58, 99, 100, 148)
Heavy metals and industrial toxicants	Altered bone metabolism and regenerative responses	Predominantly experimental, toxicological, and mechanistic evidence	Limited direct oral clinical evidence	Direct effects on oral wound healing, bone regeneration, and osseointegration remain insufficiently characterized	Hypothesis-generating	(59, 115, 116)
Occupational chemical and industrial exposures	Oral mucosal, periodontal, and salivary alterations	Mainly observational and exposure-specific human studies supported by mechanistic plausibility	Available but heterogeneous	Mixed exposures, heterogeneous occupational settings, variable exposure assessment, and substantial potential confounding	Supported but predominantly associative	(5, 25, 119, 120)
Ultraviolet radiation	Actinic cheilitis, epithelial dysplasia, and lip SCC	Clinical, epidemiological, and histopathological evidence supported by established photobiological mechanisms	Substantial and consistent	Difficulty quantifying cumulative individual exposure; variation in occupational/recreational exposure and individual susceptibility	Established	(21, 104, 105, 160, 161)
Endocrine-disrupting chemicals	Altered bone remodeling and potential periodontal/peri-implant effects	Predominantly experimental and mechanistic evidence	Limited direct oral human evidence	Heterogeneous compounds and exposure windows; scarcity of longitudinal oral-specific studies	Hypothesis-generating	(5, 22)
Microplastics and nanoplastics	Oral epithelial injury, inflammatory alterations, microbiome disruption, and impaired tissue homeostasis	Predominantly experimental and mechanistic evidence	Very limited direct clinical evidence	Lack of standardized exposure assessment, uncertain dose–response relationships, and scarcity of longitudinal oral-specific studies	Hypothesis-generating	(18, 121)
Dietary acidic exposure	Erosive tooth wear	Observational human studies, systematic reviews/meta-analyses, and experimental evidence	Substantial for specific dietary acidic exposures	Heterogeneity in dietary assessment; interaction with salivary function, gastroesophageal reflux, eating disorders, and behavioral factors	Established	(187, 188, 200, 203–206)
Dietary, behavioral, and socioeconomic determinants	Dental caries	Extensive observational and epidemiological evidence	Substantial, although the outcome is strongly multifactorial	Difficult isolation of individual determinants because of interactions among diet, fluoride exposure, oral hygiene, saliva, microbiome, and socioeconomic conditions	Established	(185, 199, 202)
Prenatal and early-life environmental exposures	Developmental defects of enamel and altered dental mineralization	Predominantly observational human evidence supported by developmental and mechanistic studies	Available but exposure-dependent	Multifactorial etiology, retrospective exposure assessment, heterogeneous diagnostic criteria, and difficulty isolating individual exposures	Supported but predominantly associative	(226–230)

Evidence interpretation represents a qualitative appraisal of evidentiary maturity within the scope of this narrative review and should not be interpreted as a formal GRADE certainty-of-evidence assessment. Classification considered the predominant study design, availability and consistency of human evidence, directness of the exposure–outcome relationship, and supporting experimental or mechanistic evidence. ***Established*** indicates relationships supported by substantial and convergent human evidence; ***supported but predominantly associative*** indicates relationships for which human evidence is available but is derived mainly from observational studies with relevant limitations to causal inference; ***emerging*** indicates relationships supported by limited human evidence together with a substantial contribution from experimental or translational findings; and ***hypothesis-generating*** indicates relationships supported predominantly by mechanistic, preclinical, or indirectly extrapolated evidence, with insufficient direct clinical confirmation. These categories reflect evidentiary maturity rather than formal estimates of effect magnitude or certainty. OPMDs, oral potentially malignant disorders; OSCC, oral squamous cell carcinoma; SCC, squamous cell carcinoma.

## 4 Discussion

The evidence synthesized in this review shows that the different components of the oral exposome are not supported by the same level of evidence. A distinction is therefore needed between exposure–outcome relationships supported by substantial human evidence and those that remain predominantly associative, emerging, or hypothesis-generating. As summarized in Table 3, differences in evidentiary maturity occur not only among environmental exposures but also among the oral outcomes attributed to the same exposure. This distinction is particularly relevant in the context of the oral exposome, where a biologically plausible mechanism does not necessarily imply that a corresponding clinical effect has been demonstrated.

Among the exposures considered, conventional tobacco smoking is supported by the most mature body of evidence. Its relationships with periodontal tissue destruction, impaired healing and implant-related complications, and oral epithelial dysplasia and carcinogenesis are supported by convergent clinical, epidemiological, and mechanistic findings (15, 112, 145, 154). Similarly, substantial human evidence supports the contribution of alcohol to oral carcinogenesis and the relationship between chronic ultraviolet exposure, actinic cheilitis, and lip squamous cell carcinoma (21, 145, 160, 161, 169). In these settings, mechanistic evidence largely complements relationships already documented in human populations rather than providing their principal basis.

The interpretation is less straightforward for several other components of the oral exposome. For air pollution and selected heavy-metal exposures, human studies support associations with periodontal, mucosal, or potentially malignant alterations, but much of the available evidence remains observational (5, 19, 148). Differences in exposure assessment, duration and intensity of exposure, co-exposures, outcome definitions, and control of confounding limit causal inference. Evidence concerning electronic cigarettes and heated tobacco products is also expanding, including studies addressing periodontal and mucosal effects, salivary alterations, and changes in the oral microbiome (16, 17, 168, 180). However, the relatively short history of widespread use, rapid evolution of devices and formulations, frequent dual use with conventional cigarettes, and limited longitudinal follow-up make long-term clinical effects more difficult to establish. These limitations are important when findings from newer tobacco-related products are compared with the much larger evidence base available for conventional smoking.

An even greater degree of caution is required for endocrine-disrupting chemicals and emerging contaminants such as microplastics and nanoplastics. In these areas, direct oral human evidence remains limited, while much of the current interpretation derives from experimental, mechanistic, or translational findings (5, 18, 22, 121). Oxidative stress, inflammatory activation, epithelial injury, altered cellular signaling, and disruption of tissue homeostasis provide biologically plausible pathways through which these exposures could affect oral tissues. Nevertheless, biological plausibility alone cannot establish a clinically relevant exposure–disease relationship. At present, these findings are more appropriately considered a basis for further investigation than evidence of established oral disease risk.

This gradient in evidence is particularly important for oral surgery, implantology, and regenerative dentistry. Conventional smoking represents the clearest example of an environmental exposure for which adverse effects on postoperative healing and implant-related outcomes are supported by clinical evidence (111, 112). By contrast, the proposed effects of air pollution, heavy metals, endocrine-disrupting chemicals, and emerging environmental contaminants on osseointegration, graft integration, or oral regenerative capacity rely to a greater extent on experimental observations, mechanistic plausibility, or evidence derived from bone and vascular biology (22, 113, 115, 116). These findings identify potentially relevant biological pathways, but they should not be interpreted as demonstrating direct causal impairment of oral regenerative procedures in exposed patients. Longitudinal clinical investigations combining objective exposure assessment with standardized surgical, regenerative, and implant-related outcomes are needed before exposure-specific clinical risks can be defined.

Against this heterogeneous evidentiary background, the oral exposome remains useful primarily as an integrative conceptual framework. The biological effects of environmental exposure on the oral cavity cannot always be adequately interpreted through a paradigm based exclusively on isolated risk factors. Environmental, behavioral, microbial, and systemic determinants may interact over time with host adaptive responses and contribute to differences in oral tissue susceptibility (10). This perspective may also help explain the considerable interindividual variability encountered in clinical practice, where individuals exposed to apparently similar environmental conditions may develop different patterns of oral disease according to host susceptibility, microbial ecology, and tissue-specific adaptive capacity (256). Importantly, however, an exposomic interpretation should not imply equivalent causal evidence for all of its components.

Previous reviews have generally addressed individual environmental exposures or specific oral diseases separately, whereas the present review brings these observations together within a common biological framework (257). Periodontal and peri-implant diseases, oral mucosal disorders, dental hard tissue alterations, impaired wound healing, and oral carcinogenesis differ substantially in etiology and clinical expression, but several of the studies considered here implicate overlapping pathways involving oxidative stress, chronic inflammation, epithelial barrier dysfunction, microbial dysbiosis, immune dysregulation, and altered regenerative responses (258, 259). These shared pathways provide a rationale for examining environmental determinants across traditional disciplinary boundaries, while the differences in evidence strength summarized in Table 3 prevent mechanistic similarity from being interpreted as equivalent clinical evidence.

Saliva, the oral microbiome, and epithelial barrier integrity are particularly relevant to this integrated interpretation because they collectively constitute the interface between external exposures and host tissues (1, 32). Their disruption may modify local responses to environmental stressors, although the relative contribution of these interconnected pathways to specific clinical outcomes remains incompletely characterized, particularly for emerging environmental determinants.

A further feature of the exposomic perspective is its emphasis on cumulative and time-dependent exposure (5). Repeated interaction with tobacco-derived products, dietary factors, occupational toxicants, air pollution, systemic inflammatory conditions, medications, and age-related biological changes may progressively influence inflammatory responses, microbial composition, epithelial integrity, vascular function, and regenerative capacity (4, 5, 260). Rather than assuming that oral diseases result from a single environmental determinant, this perspective emphasizes the potential contribution of exposure history and its interaction with host susceptibility (20). Consideration of lifetime exposure patterns may therefore provide additional context when evaluating oral disease susceptibility and treatment outcomes (261), although the relative contribution, timing, and interaction of individual exposures remain difficult to quantify.

These considerations have implications for clinical practice, but their translation should reflect the maturity of the underlying evidence. Conventional diagnostic and therapeutic approaches frequently focus on established risk factors and existing disease manifestations, whereas an exposomic perspective encourages broader consideration of the biological environment in which disease develops (7). Incorporating environmental exposure history, lifestyle characteristics, occupational conditions, systemic health, and behavioral determinants into clinical assessment may provide additional information for individualized risk evaluation and preventive planning (262). The clinical relevance of individual exposures should nevertheless be weighted according to the available evidence rather than treated uniformly. This distinction is particularly important in oral medicine, periodontology, implantology, and oral surgery, where established factors such as smoking can already inform risk assessment, whereas the clinical implications of several emerging contaminants remain to be determined. Multidisciplinary collaboration among oral healthcare professionals, public health specialists, environmental scientists, and primary care physicians may facilitate more comprehensive evaluation of exposure-related determinants (263).

From a clinical perspective, a distinction should therefore be made between applications that can already be informed by established exposure-related evidence and those that remain primarily prospective. At present, consideration of well-established factors such as conventional smoking can contribute to risk assessment in periodontal, surgical, regenerative, and implant-related care, particularly in relation to healing and treatment complications (111, 112). By contrast, the incorporation of broader exposomic profiles, environmental pollutants, emerging contaminants, salivary or microbiome-derived biomarkers, and integrated exposure signatures into individualized risk prediction is not yet supported by sufficient prospective clinical validation for routine use. Similarly, exposure-based prediction of osseointegration, graft integration, or regenerative outcomes beyond established risk factors should currently be regarded as a research direction rather than a validated clinical application. Precision oral medicine based on integrated exposomic, microbial, molecular, and host-response data therefore represents a promising future framework, but its clinical implementation will require standardized exposure assessment, validated biomarkers, longitudinal studies, and externally validated predictive models.

At the population level, the oral exposome may also provide a useful framework for preventive dentistry and public health (11). Several determinants discussed in this review, including tobacco use, dietary habits, occupational exposure, air quality, hydration status, and metabolic control, are potentially modifiable (264). Reducing harmful exposures is therefore consistent with broader preventive strategies aimed at limiting disease burden and preserving oral health. However, the strength of evidence supporting specific preventive interventions differs across exposures, and recommendations should remain proportionate to the available clinical evidence. Integrating environmental health considerations into oral healthcare may complement established preventive approaches by drawing attention to upstream determinants of disease (265), while avoiding the assumption that every putative component of the oral exposome currently has demonstrated clinical utility.

From an exposomic perspective, the COVID-19 pandemic provides an illustrative context in which infection, systemic inflammation, therapeutic exposures, changes in lifestyle and healthcare access, and host susceptibility converged to influence oral health. The reported spectrum of COVID-19-associated oral manifestations, including mucosal lesions, dysgeusia, and osteonecrosis, illustrates how alterations affecting both soft and hard oral tissues may arise within a complex network of biological, treatment-related, behavioral, and environmental determinants (266, 267). Rather than identifying a single pathway responsible for these heterogeneous manifestations, this example highlights the importance of considering interacting determinants when interpreting changes in oral homeostasis.

The present review should nevertheless be interpreted in light of several limitations. First, as a narrative review, its primary objective was to provide a broad biological and clinically oriented synthesis rather than a quantitative estimate of exposure-specific effects. No formal risk-of-bias assessment or meta-analysis was performed because the literature considered spans markedly different study designs, including experimental models, mechanistic investigations, translational studies, epidemiological research, and observational clinical studies. Second, a substantial proportion of the evidence remains observational or preclinical, limiting causal inference for many individual exposure–outcome relationships. Environmental determinants also rarely occur in isolation and frequently coexist with socioeconomic conditions, healthcare access, nutritional habits, systemic diseases, medication use, and lifestyle-related behaviors, making the independent contribution of individual exposures difficult to determine (27). Considerable heterogeneity in exposure assessment, microbiome characterization, biomarker evaluation, and clinical outcome definitions further limits comparison across studies. Finally, restricting the review to English-language publications may have resulted in the omission of relevant evidence published in other languages.

The qualitative evidence-strength categories used in Table 3 should also be interpreted within these methodological boundaries. They were introduced to distinguish comparatively mature human evidence from predominantly observational associations, emerging translational findings, and mechanistic or preclinical hypotheses, rather than to provide formal certainty-of-evidence ratings. Because no standardized risk-of-bias assessment was performed, the categories necessarily retain an element of narrative judgment and should not be interpreted as equivalent to a formal GRADE assessment. Exposure-specific systematic reviews using standardized risk-of-bias frameworks and formal certainty assessment will be needed as individual areas of the oral exposome literature become sufficiently mature.

These limitations also define priorities for future research. Advances in exposomics, multi-omics technologies, salivary biomarker profiling, microbiome analysis, metabolomics, and systems biology may improve characterization of the interactions linking environmental exposures with oral disease susceptibility (268). Particular priority should be given to longitudinal studies incorporating objective or biomarker-based exposure measurements, repeated assessment over time, standardized oral outcomes, and adequate control of major confounding factors. Such designs would help determine whether associations currently supported primarily by mechanistic or cross-sectional evidence translate into clinically meaningful effects. They would also permit investigation of exposure timing, cumulative burden, dose–response relationships, and interactions among multiple exposures, all of which are central to the exposome concept but remain insufficiently characterized in oral research.

Overall, the available literature supports the relevance of environmental exposures to oral health while also demonstrating substantial differences in evidentiary maturity across individual exposure–outcome relationships. Established relationships coexist with predominantly observational associations and with emerging areas in which mechanistic plausibility currently exceeds direct clinical evidence. Within these boundaries, the oral exposome provides a useful framework for integrating environmental exposures with host susceptibility, microbial ecology, epithelial integrity, and tissue-specific adaptive responses across oral medicine, periodontology, cariology, implantology, oral surgery, and public health. Progress in this field will depend on moving from biologically plausible associations toward longitudinal, exposure-specific, and clinically validated evidence capable of defining which components of the oral exposome have measurable implications for disease susceptibility, treatment response, and preventive care.

## 5 Conclusions

The oral exposome provides an integrative framework for examining how environmental, behavioral, microbial, and systemic determinants interact with oral tissue homeostasis across the lifespan. However, the evidence synthesized in this review demonstrates substantial heterogeneity in evidentiary maturity among individual exposure–outcome relationships. Established relationships, particularly those involving conventional tobacco smoking, alcohol-related oral carcinogenesis, ultraviolet-related lip disease, and selected dietary determinants, coexist with predominantly observational or emerging evidence concerning air pollution, occupational toxicants, electronic cigarettes, heavy metals, endocrine-disrupting chemicals, and micro- and nanoplastics.

Accordingly, mechanistic plausibility should not be interpreted as equivalent to demonstrated clinical risk. Future research should prioritize longitudinal designs, objective exposure assessment, standardized oral outcomes, and adequate control of confounding to determine which components of the oral exposome have measurable implications for disease susceptibility, treatment response, and regenerative outcomes. Within these boundaries, integrating environmental exposure history with established clinical risk assessment may contribute to more comprehensive preventive and personalized approaches to oral healthcare.
